# Gene-Specific Linear Trends Constrain Transcriptional Variability of the Toll-like Receptor Signaling

**DOI:** 10.1016/j.cels.2020.08.007

**Published:** 2020-09-23

**Authors:** James Bagnall, William Rowe, Nissrin Alachkar, James Roberts, Hazel England, Christopher Clark, Mark Platt, Dean A. Jackson, Mark Muldoon, Pawel Paszek

**Affiliations:** 1 Division of Infection, Immunity and Respiratory Medicine, School of Biological Sciences, Faculty of Biology, Medicine and Health, Manchester Academic Health Science Centre, University of Manchester, Oxford Road, Manchester M13 9PT, UK; 2 Department of Chemistry, Centre for Analytical Science, Loughborough University, Loughborough LE11 3TU, UK; 3 Synbiochem, Manchester Institute of Biotechnology, University of Manchester, Princess Street, Manchester M1 7DN, UK; 4 Cancer Research UK Manchester Institute, University of Manchester, Wilmslow Road, Manchester M20 4BX, UK; 5 Department of Mathematics, University of Manchester, Oxford Road, Manchester M13 9PL, UK

**Keywords:** cellular heterogeneity, transcriptional bursting, stochastic gene expression, toll-like receptor, single-cell transcriptomics, stochastic modeling, TNF-α, IL-1β

## Abstract

Single-cell gene expression is inherently variable, but how this variability is controlled in response to stimulation remains unclear. Here, we use single-cell RNA-seq and single-molecule mRNA counting (smFISH) to study inducible gene expression in the immune toll-like receptor system. We show that mRNA counts of tumor necrosis factor α conform to a standard stochastic switch model, while transcription of interleukin-1β involves an additional regulatory step resulting in increased heterogeneity. Despite different modes of regulation, systematic analysis of single-cell data for a range of genes demonstrates that the variability in transcript count is linearly constrained by the mean response over a range of conditions. Mathematical modeling of smFISH counts and experimental perturbation of chromatin state demonstrates that linear constraints emerge through modulation of transcriptional bursting along with gene-specific relationships. Overall, our analyses demonstrate that the variability of the inducible single-cell mRNA response is constrained by transcriptional bursting.

## Highlights

• Single-cell TNF-α and IL-1β mRNA responses are differentially controlled
• Variability of TLR-induced responses scale linearly with mean mRNA counts
• Gene-specific constraints emerge via modulation of transcriptional bursting
• Chromatin state regulates transcriptional bursting of IL-1β

## Graphical Abstract

**Figure:**
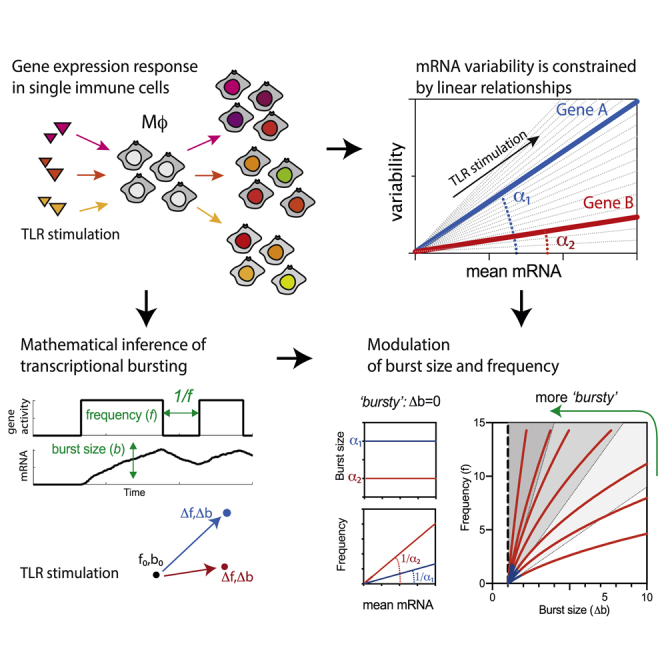

## Teaser

This study demonstrates that transcriptional variability of the toll-like receptor signaling is constrained by mean mRNA responses through gene-specific modulation of transcriptional bursting.

## Introduction

Transcription of almost all mammalian genes is regulated by transitions in their association with active RNA polymerase complexes. This often results in brief periods of transcriptional activity and stochastic bursts of mRNA output characterized by their size and frequency (Raj et al., 2006; Raj and van Oudenaarden, 2008; Suter et al., 2011). Specific gene responses may exhibit different levels of heterogeneity, arising from variations in genome architecture (Dar et al., 2012; Dey et al., 2015; Nicolas et al., 2018; Zoller et al., 2015) in concert with regulatory signaling events (Larson et al., 2013; Megaridis et al., 2018; Wong et al., 2018), through "intrinsic noise" in the stochastic process as well as extrinsic differences between cells (Elowitz et al., 2002; Hilfinger and Paulsson, 2011; Sherman et al., 2015). A recent study (Larsson et al., 2019) demonstrated that while core promoter elements control burst sizes, regulation of bursting frequency via enhancer elements defines cell-type-specific expression variability. Similarly, histone acetylation can control burst frequency, but not burst size, to regulate the circadian gene output (Nicolas et al., 2018). It is generally assumed that single-cell, and thus, population-level responses to stimulation must be tightly controlled (Paszek et al., 2010; Stelling et al., 2004), although how this is achieved in the presence of the inherent noise is not fully understood. Analyses of gene expression from reporter cells suggest a paradigm where the noise of gene expression is inversely proportional to the mean expression level (Dar et al., 2012, 2016). However, these analyses rarely involve systematic perturbation of the same gene output and have not been performed on a genome-wide scale. Consequently, there is currently no clear understanding of how the variability of specific mRNAs change as a function of the magnitude of the response to acute stimulation or general perturbation.

In order to investigate the control of cellular variability, we used the well characterized toll-like receptor signaling (TLR) system (Medzhitov, 2007). TLR represents an acute innate defense mechanism against evolutionary-conserved pathogen-associated molecular patterns and involves a coordinated production of hundreds of genes, including pro-inflammatory cytokines and chemokines (Bryant et al., 2015). The TLR effector response requires a fine balance between rapid yet robust immune activation while preventing out-of-control inflammation driving disease states (Bradley, 2008; Dinarello, 2011). Population-level studies suggest a highly constrained model, where the target gene response is subjected to a tight epigenetic and transcriptional regulation (Adamik et al., 2013; Escoubet-Lozach et al., 2011; Hao and Baltimore, 2009; Martin et al., 2020; Meissner et al., 2013; Oda and Kitano, 2006; Ramirez-Carrozzi et al., 2009; Tong et al., 2016). In contrast, at the single-cell level, TLR-dependent gene-expression responses exhibit high variability (Avraham et al., 2015; Lu et al., 2015; Shalek et al., 2013, 2014; Xue et al., 2015). This variability is thought to reflect complex transcriptional regulation, involving dynamic transcription factor (TF) signaling (Bagnall et al., 2018; Selimkhanov et al., 2014; Sung et al., 2014) as well as diverse genomic architecture (Hagai et al., 2018) and quorum licensing (Muldoon et al., 2020). For example, interferon (IFN) and tumor necrosis factor alpha (TNF-α)-mediated paracrine signals, which alter the repertoire of TF activation have been shown to regulate the heterogeneity of TLR responses (Shalek et al., 2014). However, the mechanisms by which the TLR system controls transcriptional bursting in order to regulate the heterogeneity of the target gene expression is not fully understood.

**Figure 1:**
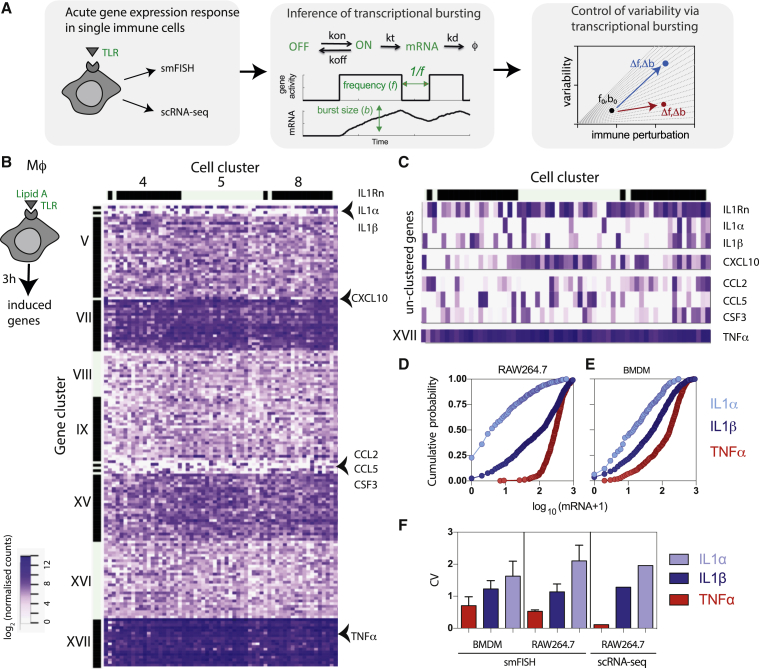
TLR4-Induced Effector Response Exhibit Differential Heterogeneity (A) Schematic representation of the data analysis pipeline: gene-by-gene single-cell expression data are systematically analyzed across a range of immune-relevant conditions to understand the modulation of transcriptional bursting characteristics and control of cellular heterogeneity. (B) scRNA-seq analysis of inducible TLR gene expression in RAW 264.7 cells stimulated with 500 ng/mL of lipid A for 3 h. Heatmap displaying normalized transcript levels of high confidence genes upregulated in response to lipid A stimulation. Major gene clusters are shown in roman numerals, cell clusters depicted with Arabic numerals. Arrowheads highlight specific unclustered genes as well as TNF-α. (C) Heatmap of unclustered gene set from (B). Also shown is the heatmap of *TNF-α* expression. (D) smFISH analysis of the cumulative probability distribution of *IL-1α*, *IL-1β,* and *TNF-α* mRNA expression in RAW 264.7 cells stimulated with 500 ng/mL of lipid A for 3 h. Count data expressed as log_10_(mRNA+1) from 447, 718, and 356 cells, pooled across at least three experimental replicates, respectively. (E) Cumulative probability distribution of mRNA counts in BMDMs (stimulated as in D). Shown is the analysis of 447, 732, and 322 cells for *IL-1α*, *IL-1β,* and *TNF-α*, pooled across at least three experimental replicates, respectively. (F) Variability of *IL-1α*, *IL-1β,* and *TNF-α* expression in scRNA-seq and smFISH data. Shown is the coefficient of variation (CV) calculated for respective genes across datasets, with SDs between biological replicates (when available).

In this study, in order to uncover mechanisms that control gene-expression variability in the TLR system, we used single-molecule mRNA and single-cell RNA-seq (scRNA-seq) data obtained via systematic perturbation of individual gene outputs across immune-relevant conditions (Figure 1A). We specifically measured and mathematically modeled mRNA count distributions of TLR-dependent interleukin-1β (IL-1β) and TNF-α. We demonstrated that in response to 14 different TLR conditions the variability of the individual mRNA response can be empirically described by a linear function of the mean. These linear relationships are also present in 204 TLR-regulated genes in the scRNA-seq dataset from bone marrow dendritic cells (BMDCs) (Shalek et al., 2014). In the context of the stochastic telegraph model, we determined the ways in which the linear relationships constrain the underlying bursting characteristics. Theoretical predictions were subsequently validated by the analysis of TNF-α and IL-1β smFISH counts, including additional experimental perturbation of the chromatin state.

## Results

### Expression of IL-1β and TNF-α mRNAs Exhibit Different Levels of Cellular Heterogeneity

To obtain insights into the control of cellular variability in the TLR system, we first characterized gene-expression patterns in innate immune macrophages by single-cell transcriptomics (Figure 1A). We generated single-cell RNA-seq libraries using the C1 Auto Prep System (Fluidigm C1) using an established RAW 264.7 macrophage cell line (Bagnall et al., 2018; Cheng et al., 2015; Sung et al., 2014) stimulated with lipid A for 3 h (the main cytotoxic component of TLR4 agonist lipopolysaccharides [LPS]; Raetz et al., 2007). After mapping and normalization (Figure S1), high-confidence genes (171 genes with higher expression and, hence, lower technical variance; Figure S1F), which were found to be regulated by lipid A in a previous population-level study (Bagnall et al., 2015), were clustered using an unsupervised affinity propagation method (Frey and Dueck, 2007). The analysis yielded 7 distinct major gene clusters and 3 uniform cell clusters (Figures 1B, S2A, and S2B; Table S1). For example, cluster XVII comprised 18 most abundant genes, including the effector cytokine *TNF-α* in addition to chemokines *Ccl9* and *Cxcl2*. Notably, we found a set of 10 genes that failed to cluster (referred herein as the “unclustered gene set”; Figure 1C). These included the pro-inflammatory inflammasome-associated cytokines *IL-1α* and *IL-1β* (Martinon et al., 2002) in addition to *IL-1rn* (interleukin-1 receptor antagonist), which are co-located in mouse and human genomes (Smith et al., 2004; Taylor et al., 2002). Other unclustered genes encoded chemokines: *Cxcl10*, *Ccl2,* and *Ccl5* and a pro-survival colony-stimulating factor *Csf3*, a ligand *Jag1* (Jagged1) (Hu et al., 2008), protein kinase (Plk2), a regulator of TNF-α secretion (Schwarz et al., 2014), and a membrane DC-stamp protein involved in cell fusion (Yagi et al., 2005).

Unclustered genes exhibited more variability than genes belonging to major clusters, while housekeeping genes were the most homogeneous (Figure S2C). Higher variation was not solely associated with technical noise as some major cluster genes have a higher number of mapped reads than the housekeeping genes (for example, clusters XVII and VII; Figure S2D). Similarly, unclustered genes do not have appreciably lower numbers of mapped reads than other genes and, indeed, have more in many cases. Expression heterogeneity may be related to physical gene properties (Hagai et al., 2018; Larsson et al., 2019), for instance, levels of transcriptional bursting have been linked to the presence of TATA boxes within gene promoters (Zoller et al., 2015). Indeed, we observe that unclustered genes exhibit significant enrichment of TATA sites in the promoter regions as well as a strong association between the transcript synthesis rate and variation (Figure S3).

We used quantitative smFISH to validate and accurately quantify expression patterns of *TNF-α*, *IL-1α,* and *IL-1β* mRNA in single cells (Figures 1D, 1E, and S4A–S4D). The average expression of *IL-1β* (± standard deviation, SD) was 215 ± 230 mRNA molecules for count data combined across all replicates. 50% of RAW 264.7 cells expressed more than 100 *IL-1β* mRNA molecules (with some expressing up to 1,000 molecules), while 20% of cells expressed <10 mRNA molecules (see Figure 1D for the cumulative probability function and Figure S4B for a histogram of smFISH counts). *TNF-α*, a cytokine that plays fundamental but distinct roles during infection (Adamik et al., 2013; Falvo et al., 2010), exhibited a similar level of expression on an average (255 ±144 mRNA molecules), but 90% of cells expressed more than 100 mRNA molecules (evident of reduced variability). We confirmed that the heterogeneous *IL-1β* expression patterns were seen in primary bone-marrow-derived macrophages (BMDM) (Figures 1E, S4C, and S4D), with correlated protein expression (Figures S4E–S4H) as well as in LPS-stimulated dendritic cells (Shalek et al., 2014) (Figure S5). There was also a good agreement between smFISH counts and our scRNA-seq study displaying similar levels of noise (Figure 1F). Overall, these analyses demonstrate conserved variability in the TLR system across cell types and suggest that *IL-1β* and *TNF-α* expression may have different modes of regulation.

### Mathematical Modeling of mRNA Count Data Distinguishes Regulatory Modes

**Figure 2:**
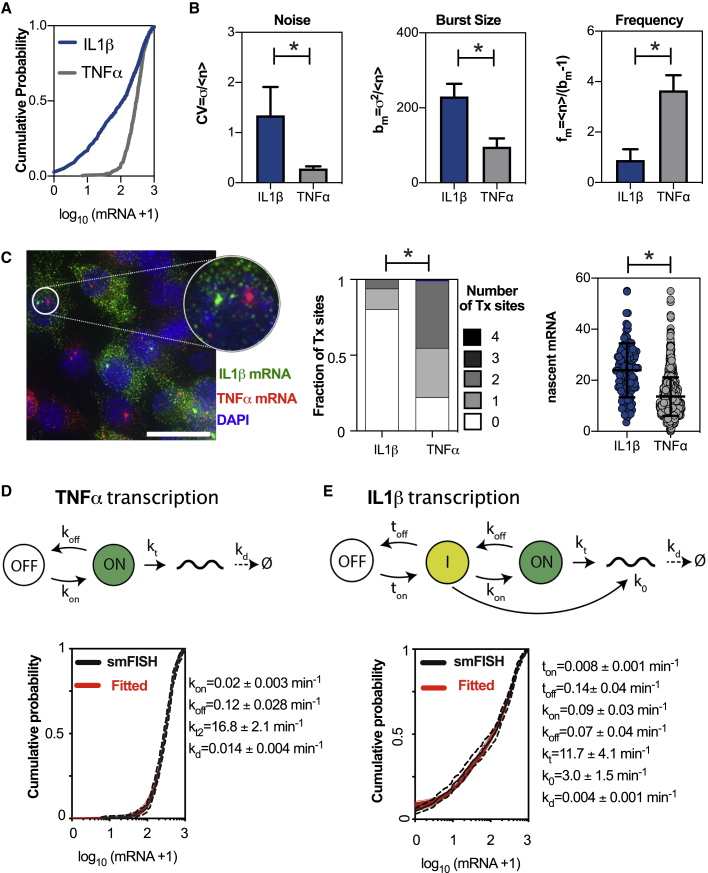
Mathematical Modeling Reveals Differential Control of *TNF-α* and *IL-1β* Transcription (A) Differential expression of *IL-1β* and *TNF-α* mRNA. Shown is the cumulative distribution function of mRNA counts in RAW 264.7 macrophages stimulated with 500 ng/mL of lipid A for 3 h. A total of 718 cells were measured for *IL1β*, and 356 for *TNF-α*, and pooled across at least three smFISH experiments, respectively, and expressed as log_10_(mRNA+1). (B) Characteristics of single-cell mRNA expression. Shown is the CV, burst size (*b*_*m*_), and frequency (*f*_*m*_) calculated based on moments of the mRNA count data from (A) (expressed as mean ± SD from experimental replicates). “^∗^” denotes a result of a two-sample Mann-Whitney U test between groups (p < 0.01). (C) Distribution of transcription sites is gene dependent. (Left) de-convolved wide-field microscopy image of cells with *TNF-α* and *IL-1β* smFISH, revealing Tx through an aggregation of multiple mRNA molecules in the nucleus (insert). Scale bar represents 5 μm. (Middle) the fraction of cells with 0–4 Tx calculated from (A). “^∗^” denotes a result of the Fisher exact test (p < 0.05) for difference in the Tx site distributions. (Right) the number of nascent mRNA per Tx. Shown are individual Tx site data, together with the mean and SD of the pooled distribution. “^∗^” denotes a result of a two-sample Mann-Whitney U test between groups (p < 0.01). (D) *TNF-α* transcription conforms to a one-step stochastic model. The comparison between measured and fitted *TNF-α* mRNA distributions at 3 h after 500 ng/mL lipid A treatment. In black: a Kaplan-Meier estimator of the measured cumulative distribution functions (CDF) (with 95% confidence intervals); and in red: a family of 50 models fitted to the data. Fitted parameter values (means ± SD) listed on the right. (E) *IL-1β* transcription conforms to a two-step stochastic model. The comparison between measured and fitted *IL-1β* mRNA distributions at 3 h after 500 ng/mL lipid A treatment for the depicted model. In black: Kaplan-Meier estimator of measured CDF (with 95% confidence intervals); and in red: family of 50 models fitted to the data. Fitted parameter values (means ± SD) listed on the right.

The heterogeneity of gene expression has typically been characterized in terms of transcriptional bursting, i.e., the process of intermittent gene activation (So et al., 2011). The characteristics of the transcriptional burst process, such as burst size and burst frequency, are defined as the average number of mRNA produced per gene activation event and the frequency of gene activation events, respectively (Nicolas et al., 2017). We first used the sample variance *σ*^2^ and the mean *μ* of the mRNA distribution to compute an approximate burst size *b*_*m*_*=σ*^*2*^*/μ* (i.e., the Fano factor) and burst frequency *f*_*m*_*=μ/(b*_*m*_*−1)* (Nicolas et al., 2017; Raj et al., 2006; Suter et al., 2011) in order to understand the difference in *TNF-α* and *IL-1β* regulation. In general, these quantities (referred to here as “moment estimators”) are often used to describe “burstiness” by quantitatively capturing departures from “non-bursty” (Poissonian) mRNA production (for which *b*_*m*_ = *1 and f*_*m*_ *= ∞*) (Nicolas et al., 2017; So et al., 2011; Wong et al., 2018) (see Figure S6 for general applicability of the moment estimators). Analysis of the noise level (*CV = σ/μ*), burst size (*b*_*m*_), and burst frequency (*f*_*m*_) based on the moments of the smFISH count distribution (Figure 2A) showed that *IL-1β* exhibits more burstiness, i.e., larger relative burst sizes and lower frequency compared with that of the more homogeneous *TNF-α* (Figure 2B). On-going *IL-1β* transcription, visualized via bright nuclear spots of fluorescence in the smFISH images (Femino et al., 1998; Skinner et al., 2016; Zenklusen et al., 2008), was evident in only 20% of cells (Figure 2C). In contrast, up to 75% of cells possessed at least one *TNF-α* transcription site (Tx). There was even an indication of *TNF-α* transcription immediately prior to cell division by the presence of >2 Tx sites in a subset of cells. We also observed more nascent mRNA associated with Tx sites for *IL-1β* than *TNF-α*. This is consistent with more infrequent but larger mRNA bursts in comparison to *TNF-α*. These characteristics were conserved across different doses of lipid A stimulation (including in BMDMs; Figures S7 and S8) as well as time (Figure S9), confirming that *IL-1β* and *TNF-α* exhibited distinct modes of transcriptional bursting.

The classical mathematical description of mRNA production involves a one-step stochastic telegraph model, where gene activity switches randomly between “off” and “on” states, with only the latter being permissive for mRNA transcription (Raj et al., 2006; Skinner et al., 2016; Suter et al., 2011; Zenklusen et al., 2008). The associated kinetic parameters include gene activation switching “on” and “off” rates (*k*_*on*_ and *k*_*off*_, respectively) as well as rates of mRNA transcription and degradation (*k*_*t*_ and *k*_*d*_, respectively); Figure 2D. In this case, bursting parameters are directly related to the kinetic parameters of transcription (Nicolas et al., 2018). The steady-state burst size is defined as *b*_*k =*_ *k*_*t*_*/k*_*off*_, while bursting frequency is given by *f*_*k*_ *= 2k*_*on*_*k*_*off*_*/ (k*_*on+*_*k*_*off*_*)/k*_*d*_ (these are referred herein as “kinetic estimators”; Figure S6). In order to apply these stochastic models, we first investigated the sources of the variability in smFISH count data, which could either involve intrinsic stochastic fluctuations (i.e., on-off switching) or extrinsic cell-to-cell differences (Elowitz et al., 2002; Hilfinger and Paulsson, 2011; Sherman et al., 2015). We previously found a correlation between the cell size and mRNA level consistent with an extrinsic noise component (Bagnall et al., 2018), but this relationship did not affect mRNA distributions (when compared with cell size-normalized distributions) and only explained up to 7% of the data (as assessed by a correlation coefficient of a linear fit; Figure S10). Furthermore, smFISH counts exhibited a key intrinsic noise property, where noise decreased monotonically (Taniguchi et al., 2010) with mean expression, rather than approaching a plateau (Figure S11A). A formal noise decomposition of the *TNF-α* and *IL-1β* dose-response count data (Rhee et al., 2014) showed a dominant contribution from the intrinsic noise with an extrinsic noise component (Figure S11B). The latter is consistent with the extrinsic variability due to shared TLR signaling machinery, for example, signaling dynamics (Muldoon et al., 2020; Wong et al., 2018, 2019).

Given the dominant role of intrinsic noise, we, therefore, used a genetic algorithm to fit a family of one-step models (resulting in 50 kinetic parameter sets) to smFISH count distributions using biological constraints on parameter values (see STAR Methods). We found that the one-step model was able to recapitulate the measured *TNF-α* mRNA distribution in RAW 264.7 cells with an average gene switching “on” rate of *k*_*on*_ = 0.02 min^−1^ (i.e., equivalent to 50 min “off” time, *1/k*_*on*_) and a switching “off” rate of *k*_*off*_ = 0.12 min^−1^ (i.e., equivalent to 8.3 min “on” time, *1/k*_*off*_; Figure 2D). The average transcription rate of 16.8 ± 2 mRNA/min was consistent with the range previously reported for other highly inducible mammalian gene products (Molina et al., 2013; Schwanhäusser et al., 2011; Skinner et al., 2016; Suter et al., 2011) and was inversely correlated with the degradation rate (Figure S11C). We then used the one-step model to fit the distribution of *IL-1β* mRNA counts (Figure S11D), assuming a longer half-life in comparison to *TNF-α* (Hao and Baltimore, 2009). We found that the model failed to recapitulate the smFISH distribution, especially for mRNA counts below 100 molecules. We, therefore, considered more complex model structures that incorporate an additional constitutive initiation event, or additional regulatory step (equivalent to promoter cycling; Harper et al., 2011; Zoller et al., 2015), consistent with either chromatin remodeling or combinatorial TF binding driving a single transcription rate (Figure S11D models 2 and 3). These models were also unable to fit the observed data. Analysis of combined architectures suggested a model (Figures 2E and S11E) in which sequential activation and two transcription rates were required to recapitulate the entire range of mRNA counts. The first step was characterized by a small gene switching “on” rate *t*_*on*_ = 0.008 min^−1^ (equivalent of 125 min “off” time) and a low transcription output (*k*_*0*_ = 3 ±1.5 mRNA/min); in contrast, the second step was rapid *k*_*on*_ = 0.09 min^−1^ (11 min “off” time) resulting in a high transcriptional output (*k*_*t*_ = 11.7 ± 4.1 mRNA/min) (see Figure S11F for the comparison between individual on-off and transcription rates in the fitted family of models). During transcriptional activation, the first slow step is permissive for a second activation event resulting in a larger burst size and lower bursting frequency in the model, as compared with those for *TNF-α* (see Figure S11G for the estimates of the burst size and frequency from the models and Figure S11H for sensitivity analyses of model structures).

### Transcriptional Heterogeneity Is Constrained by Gene-Specific Linear Trends

**Figure 3:**
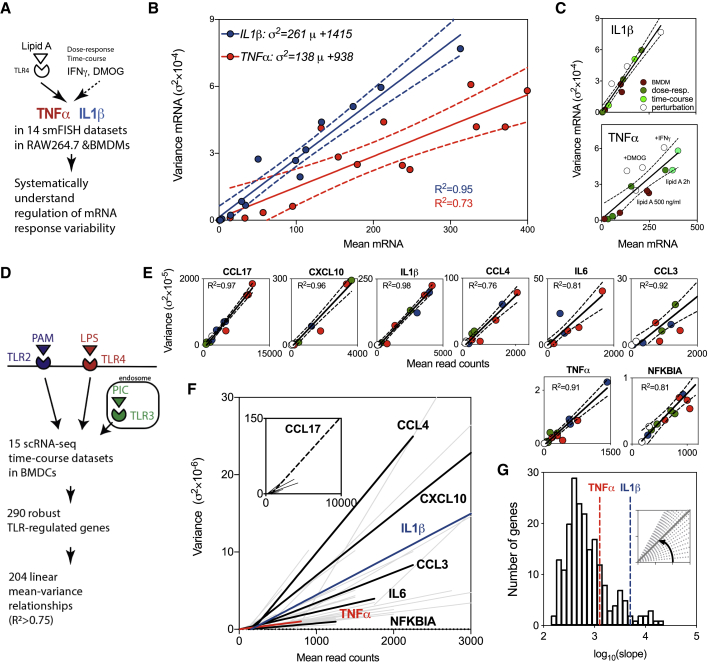
Single-Cell Expression is Constrained by Gene-Specific Linear Trends (A) Analysis of single-cell variability in *TNF-α* and *IL-1β* mRNA expression across 14 smFISH measurements; dose response in RAW 264.7 and BMDM cells; time course in RAW 264.7 as well as DMOG and IFNγ co-stimulation in RAW 264.7 cells. (B) Mean-variance relationship obtained for smFISH data for *IL-1β* and *TNF-α*. Shown is the fitted regression line (with 95% confidence intervals in broken lines), together with individual data points. Coefficient of determination depicted with *R*^*2*^ and color coded. Fitted equations displayed on the graph. (C) Visualization of samples across data in (B). Individual data points colored and labeled: green- RAW 264.7 dose-response data; light green, RAW 264.7 time course data; open circles, RAW 264.7, DMOG, and IFNγ co-stimulation data; and brown, BMDM dose-response. (D) Inference of mean-variance relationships from the scRNA-seq data from (Shalek et al., 2014). BMDCs either untreated or stimulated with TLR2, 3, and 4 ligands for 1, 2, 4, or 6 h. For each TLR-dependent gene in the dataset, mean and variance of read count expression across all conditions are fitted using robust linear regression. (E) Analysis of mean-variance relationships in selected TLR-induced genes. Shown are the fitted linear regression lines (with 95% confidence intervals) for highlighted genes from (Shalek et al., 2014). Different TLR treatments color coded as in (D) (open circles, untreated controls). Coefficient of determination depicted with *R*^*2*^. (F) Linear mean-variance regression trends for 204 high-confidence genes inferred from (Shalek et al., 2014). Highlighted genes depicted in black, trends for *IL-1β* and *TNF-α* in blue and red, respectively. (G) Distribution of fitted regression slopes from (F) (in log_10_). Slopes for *IL-1β* and *TN-Fα* regression fits highlighted in blue and red lines, respectively.

While our analyses demonstrate different levels of single-cell gene-expression heterogeneity in the TLR system, a fundamental question remains whether, and how, this heterogeneity is altered in response to stimulation or perturbation (Dar et al., 2012, 2016). In order to address this question, we systematically analyzed all smFISH datasets (Figure 3A) comprising the dose- and time-dependent responses in RAW 264.7 cells and BMDMs to lipid A stimulation (Figures S7–S9) as well as additional immunologically relevant conditions (Figure S12). We used a 24-h interferon γ (IFNγ) pretreatment before lipid A stimulation, to mimic Signal Transducer and Activator of Transcription 1 (STAT1)-dependent inflammatory signaling (Bryant et al., 2015), which reduced *IL-1β* and increased *TNF-α* mRNA production (in comparison to stimulation with lipid A alone; Figures S12A and S12B). In turn, pretreatment with prolyl hydroxylase inhibitor dimethyloxalylglycine (DMOG), a pharmacological mimic of Hypoxia Inducible Factor 1α (HIF1α)-dependent hypoxia (Bagnall et al., 2014), resulted in an elevated expression of both *IL-1β* and *TNF-α* mRNA. When all smFISH datasets were examined collectively, we found that the gene-expression variability (represented as the variance of smFISH counts) across experimental conditions was constrained by the corresponding mean of the mRNA counts (Figure 3B). The larger heterogeneity in *IL-1β* expression was reflected in a significant increase in the gradient of the mean-variance relationship (defined as a slope of the fitted regression line) than that of *TNF-α* (p value 0.00019). While some individual conditions showed departures from the fitted linear relationships (arguably more for *TNF-α* than *IL-1β*; Figure 3C), both fits were characterized by a high coefficient of determination (*R*^*2*^ *= 0.97* and *0.83*, for *IL-1β* and *TNF-α*, respectively). The fitted relationships appear to have positive intercepts, which is perhaps indicative of the limited sample size and might reflect measurement noise, therefore, we treat those as empirical relationships.

In order to establish this relationship in diverse cell types and in more genes, we took advantage of published single-cell transcriptomics data from BMDCs (Shalek et al., 2014); Figure 3D. These data included 15 scRNA-seq (each with up to 96 individual cells) time course measurements (at 0, 1, 2, 4, and 6 h) of acute responses to PAM (synthetic mimic of bacterial lipopeptides upstream of TLR2), PIC (viral-like double-stranded RNA for TLR3), and LPS ( a component of Gram-negative bacteria upstream of TLR4), referred herein as the core TLR dataset. TLR pathways share common regulatory mechanisms, yet, induce distinct gene-expression patterns (Medzhitov, 2007). For example, the expression of *TNF-α* is maintained in response to PAM but is transient in response to PIC over the 6-h period (Figures S13A and S13B). However, in agreement with our smFISH data, we found that the mean and variance of *TNF-α* read counts exhibit a close linear relationship (Figure S13C; coefficient of determination *R*^*2*^ *= 0.91*). Subsequently, we considered 290 genes that were robustly induced by LPS stimulation in the dataset, revealing 204 genes that are described by linear trends with high confidence (as defined by *R*^2^ >*0.75*; see Figure 3E for examples of specific genes and Figure 3F for the fitted relationships; Table S3 for all gene-by-gene fits). The previously observed trends in *IL-1β* and *TNF-α* expression were also present in the BMDC dataset (Figure 3F).

These analyses demonstrate that (1) the variability of mRNA expression can be empirically described by a linear function of the mean response; (2) the gene-specific variability can be defined by the slope of the regression line, constituting a spectrum at the genome level (Figure 3G). High variability genes include chemokines and cytokines, such as *CCL17*, *CCL3*, as well as *IL-1α* and *β*, while others, such as *TNF-α* (and *NFKBIA*, an inhibitor of NF-κB signaling) exhibit more homogeneous responses; (3) response patterns were shared among different TLR ligands and no difference between treatment-specific trends were found; (4) linear relationships were generally maintained under signaling perturbation involving Golgi inhibition and in Interferon-alpha receptr chain alpha (INFAR1), Tumour necrosis factor receptor 1 (TNFR), and STAT1 knockout cells (Shalek et al., 2014) (see Table S4 for gene-by-gene fits). However, the regression fit was altered in a subset of genes (as assessed by the analysis of regression slopes in the core TLR and perturbation datasets; Figures S13D–S13F), which suggests that these relationships can be regulated.

### Linear Constraints Define Properties of Transcriptional Bursting

**Figure 4:**
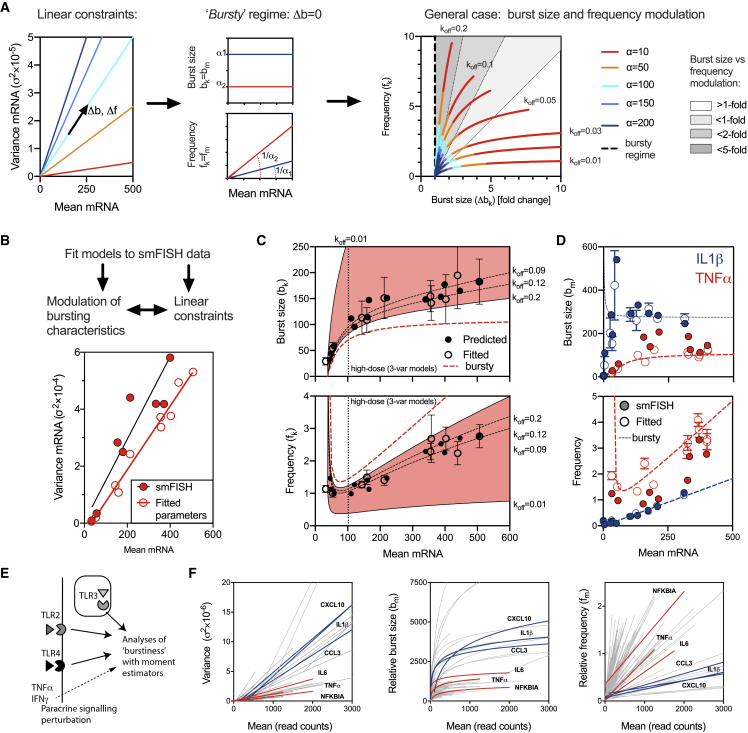
Linear Constraints Define Properties of Transcriptional Bursting (A) Reciprocal relationship between burst size and frequency. (Left) a set of considered hypothetical genes characterized by different mean-variance slope *α* (such that *σ*^*2*^*= αμ*). (Middle) frequency modulation and constant burst size in the bursty regime. (Right) concurrent burst size and frequency modulation as a function of *k*_*off*_. Calculations performed using Equation 6 for the biologically plausible set of gene activity switching rates, *k*_*off*_*< 0.2 min*^*−1*^ and *k*_*on*_*< 0.1 min*^*−1*^; *k*_*d*_*= 0.014 min*^*−1*^; *k*_*t*_*< 30 min*^*−1*^; and μ < 500. Shown are relative frequency and burst size changes (Δb_k_) over the corresponding range of the mean mRNA, calculated for each α for *k*_*off*_*= 0.01, 0.02, 0.03, 0.05, 0.075, 0.1, 0.2 min*^*−1*^, respectively. In a broken line moment estimator (i.e., bursty regime), shaded are regions corresponding to 1-fold, 2-fold, and 5-fold burst sizes versus frequency modulation. (B) Variability of the TNF-α expression across data in RAW 264.7 macrophages (dose response, time course, as well as IFNγ, IFNγ+lipid A, and DMOG+lipid A perturbation). Displayed is the relationship between sample mean and variance of individual smFISH count data (full red circles) and steady-state mean and variance (open red circles) based on fitted parameter values (Figure S17). Model outputs calculated for a family of 50 models fitted to each data point. Regression lines fitted to smFISH counts (depicted in black) and steady-state mean and variance calculated for fitted model parameters (depicted in red). (C) Burst size and frequency modulation of the *TNF-α* expression. Shown in red are regions calculated for the fitted *σ*^*2*^*= 113 μ-4249* relationship for biologically plausible set of gene activity switching rates: *k*_*off*_*< 0.2 min*^*−1*^, and *k*_*on*_*< 0.1 min*^*−1*^; and *k*_*d*_*= 0.014 min*^*−1*^, and *k*_*t*_*< 30 min*^*-1*^. Highlighted broken lines correspond to burst size and frequency changes corresponding to *k*_*off*_*= 0.01, 0.09, 0.12, 0.2 min*^*−1*^. Predicted burst sizes and burst frequencies depicted in black circles (using Equation 7 and fitted *k*_*on*_*/k*_*off*_ and *k*_*d*_ rates, from Figure S17), in open circles steady-state estimates using fitted parameter values. The broken red line shows a predicted behavior in the bursty regime based on the fitted regression line. Horizontal dotted line marks a subset of data corresponding to the high-dose lipid A conditions (3-variable model fits; Figure S17). (D) Burstiness of the *IL-1β* and *TNF-α* mRNA expression. Shown are moments estimates of burst size and frequency for smFISH counts (full circles) and fitted model distributions (open circles, in blue and red for *IL-1β* and *TNF-α*, respectively) for data in RAW 264.7 macrophages (dose response, time course, as well as TSA, IFNγ, and DMOG perturbation; Figures S17 and S18). In broken red and blue lines is the predicted behavior in the bursty regime, based on the regression lines for fitted models for *TNF-α* (from B) and *IL-1β* (from Figure S18C), respectively. (E) Schematic representation of the combined (core TLR and paracrine signaling perturbation) scRNA-seq datasets from (Shalek et al., 2014). (F) Burstiness of TLR-induced genes. Shown are relationships for the variance, relative burst size (*b*_*m*_), and relative frequency (*f*_*m*_) as function on the mean read count inferred from the combined core TLR and perturbation dataset from (Shalek et al., 2014). Displayed are 204, 180, and 132 relationships for variance, relative frequency, and relative burst size (defined based on the coefficient of determination *R*^*2 >*^*0.75, R*^*2 >*^*0.7* and *R*^*2*^*> 0.5*, respectively) inferred using robust linear regression (with semi-log transformation for relative burst size). Individual high and low heterogeneity gene fits color coded and labeled.

Previous studies suggest a paradigm where transcriptional bursting constrains stochastic gene-expression programs (Dar et al., 2016; Sanchez and Golding, 2013). The existence of an empirical linear relationship between the mean and variance of the single-cell mRNA response (Figure 3) provides insight into the regulation of transcriptional bursting. We used the steady-state approximation for the mRNA moments in the one-step model (Peccoud and Ycart, 1995; Paszek, 2007; Shahrezaei and Swain, 2008) and derived theoretical relationships between model parameters for which the $\sigma^{2}=\alpha \mu$ relationship holds (Figures 4A, S14, and S15; STAR Methods for derivation and discussion). First, we considered the case of the “bursty” gene-expression regime, i.e., *k*_*off*_ *≫ k*_*on*_, when transcription occurs in short and infrequent bursts. Under these conditions, we theoretically predicted that bursting characteristics are predetermined by the empirical mean-variance relationship: (1) burst size is necessarily constant (and equal to the slope of the mean-variance line) over the range of the mean mRNA response (i.e., burst size *b*_*k*_ *= α-1*); (2) changes of gene expression are controlled solely by frequency modulation [i.e., *f*_*k*_ *= μ/(α-1)*]; and (3) there is a reciprocal relationship between the burst size and frequency, as the burst frequency is proportional to the inverse of the burst size (*1/α*). Therefore, the larger the burst size, the lower the frequency of gene expression (and vice versa) to maintain a constant mean-variance relationship. In a general case, our derivations show that both burst size and frequency may undergo modulation as the mean mRNA expression varies. The relative contribution of the burst size and frequency modulation is related to the *k*_*off*_ value (or *k*_*off*_*/k*_*on*_ ratio; Figure S15). For a range of biologically plausible parameter values (*k*_*off*_ *< 0.2 min*^*−1*^ and *k*_*on*_ *< 0.1 min*^*−1*^, while *k*_*t*_ *< 30 min*^*−1*^), the higher the *k*_*off*_ (or *k*_*off*_*/k*_*on*_ ratio), the smaller are the changes of the burst size in comparison to the changes of frequency (see Figure 4A for a set of putative genes with different levels of variability defined via slope α). For example, for *k*_*off*_ > 0.1 (and thus, relatively close to a bursty regime in the considered parameter ranges), we find 2-fold more changes of the burst frequency than that of the burst size (and 5-fold more for highly variable genes, i.e., α < 100). In turn, *k*_*off*_ < 0.02 resulted in a dominant burst size modulation (especially for low variability genes).

In order to validate our theoretical predictions, we inferred bursting characteristics in our smFISH data across different immune-relevant conditions. First, using data from RAW 264.7 cells (to avoid cell-type differences), we fitted one-step models to measured *TNF-α* distributions. Consistent with the intrinsic noise model (Elowitz et al., 2002; Hilfinger and Paulsson, 2011; Sherman et al., 2015), *TNF-α* counts across all conditions fitted negative binomial distributions (Figure S16). Initially, we assumed a common half-life across all conditions (using *k*_*d*_ = *0.014* mRNA/min estimated for the high-dose 500 ng/mL lipid A treatment; Figure 2), while fitting three remaining parameters (*k*_*on*_, *k*_*off*_, and *k*_*t*_; Figure S17). Later, all four kinetic parameters were refitted for a subset of conditions corresponding to lower lipid A doses (and thus, shorter mRNA half-life; Hao and Baltimore, 2009). Models summarized in terms of the mean-variance relationship (using fitted parameters to calculate moments; Figure 4B) captured most of the variability present in smFISH count data (with an exception of DMOG, which was subsequently not considered; Figure S17). Subsequently, we calculated the burst size and frequency changes along the fitted linear relationship (Figure 4C). Bursting characteristics were either obtained directly from fitted parameter values (using kinetic estimators) or predicted from the fitted regression line based on the fitted *k*_*off*_ and *k*_*on*_ rates (see STAR Methods for derivation and discussion of a general case of mean-variance relationships with a non-zero intercept). Both approaches demonstrate a concurrent modulation of *TNF-α* expression via the burst size and frequency as a function of mean mRNA expression. The burst size increased monotonically from ~25 to ~150 molecules across all conditions, while the burst frequency changed between 1 and 3 (with a minimum predicted for the case of a linear fit with a non-zero intercept; Figure S15E). When considering only the subset of conditions for the high-dose lipid A responses (mean mRNA > 100 and *k*_*d*_ *= 0.014* mRNA/min), the changes of the burst size were limited to <2-fold. In this case, the frequency modulation becomes dominant in agreement with the theoretical prediction (bursty regime shown by the broken red line; Figure 4C). Analysis of fitted parameters demonstrates that the modulation of bursting characteristics across the mean expression was due to an increase in the “on” rate, and a concurrent decrease in the “off” rate (Figure S17C).

Our analyses predict a link between the level of expression variability and bursting characteristics, i.e., increased variability results in increased burst size and lower burst frequency (Figure 4A). Therefore, to compare gene-specific characteristics we fitted *IL-1β* smFISH count data in RAW 264.7 cells using the previously developed two-step model (Figure S18). Given the multistep structure of the *IL-1β* model, we reverted to moment estimators (Nicolas et al., 2017; So et al., 2011). In agreement with our modeling predictions, the higher variability of *IL-1β* expression is associated with quantitatively larger burst size and lower frequency (obtained via moment estimators) than that of *TNF-α* (see Figure 4D for relationships using fitted models and smFISH count data in RAW 264.7 cells- Figures S19A and S19B for analysis of all smFISH counts). In a general case, both burst size and frequency may undergo modulation, which is evident from the analysis of the *TNF-α* regulation (Figure 4C). Our analyses predict that the contribution of the burst size modulation decreases as the system converges to the bursty regime. *IL1β* transcription exhibits more “bursty” expression in comparison to *TNFα* (Figure 2, with *t*_*off*_*/t*_*on*_ *~18* for *IL1β* in the permissive step and *k*_*off*_*/k*_*on*_*~6* for *TNFα*). We find evidence for more a dominant frequency modulation of *IL1β* expression when compared with transcriptional bursting characteristics inferred for *TNFα* (at least for high mRNA expression; Figure 4D). In agreement, the burst size of *IL-1β* mRNA production remained constant for a wide range of expression (except for small means; see STAR Methods for discussion of mean-variance with a non-zero intercept). Consistently, the fitted parameter values exhibit changes in the gene activity switching “on” rates (corresponding to both regulatory steps) over the whole range of *IL-1β* mRNA responses (Figure S18D).

Finally, we used the scRNA-seq dataset in BMDCs (Shalek et al., 2014) to gain insights into the modulation of bursting characteristics in 323 robustly expressed TLR-dependent genes (Figures 4E, 4F, and S19B–S19F). We did not make any assumptions about the transcriptional regime (since fitting models to scRNA-seq dataset was not possible due to the lack of absolute quantification in the sequencing protocol; Shalek et al., 2014), but instead, we used regression analyses to infer changes of relative burst size and frequency (described by moment estimators) across gene-specific linear relationships (see Figure S19C for inference of bursting characteristics for *TNF-α*; Tables S3, S4, and S5 for gene-by-gene visualization, including a comparison between core TLR and perturbation datasets). Despite the inherent variability of the scRNA-seq data (which was validated by remapping a subset of data; Figure S20), we found quantitative changes of burstiness across >130 individual genes consistent with relative burst size and frequency modulation (Figure 4F). By comparison of independently fitted relationships, we also found that the evidence for the predicted reciprocal bursting characteristics, including a negative correlation between the burst size and frequency as well as the correlations with the slope of the fitted mean-variance relationships are present in the dataset (Figure S19F).

### Chromatin Regulates *IL-1β* Expression via Modulation of Bursting Characteristics

Given the role of modulation of transcriptional bursting in the control of the inducible single-cell gene-expression variability, we sought to investigate the underlying mechanism. Previous work indicated the involvement of TF signaling, including that of the nuclear factor κB (NF-κB) in the TLR system in the context of chromatin regulation (Larson et al., 2013; Nicolas et al., 2018; Wong et al., 2018). We, therefore, turned our attention to *IL-1β* transcription, the two-step structure of which suggests an influence of the chromatin state. We observed a highly correlated biphasic mRNA response between *IL-1β* and *IL-1α*, whose genes are located in a single gene cluster in the mouse and human genome (Smith et al., 2004; Taylor et al., 2002), but not *TNF-α* (Figure S21A). We also observed a significant correlation between the presence of *IL-1β* and *IL1α* (but not *TNF-α*) active transcription sites (Figure S21B). We found that the transcription of *IL-1β* and *IL-1α* not only coincided temporally but also spatially, as a significant number of Tx sites co-localized, Figure S21C). Presumably, these genes sharing a local chromatin structure show a high propensity to be transcribed within a common transcription factory (Jackson, 2003).

**Figure 5:**
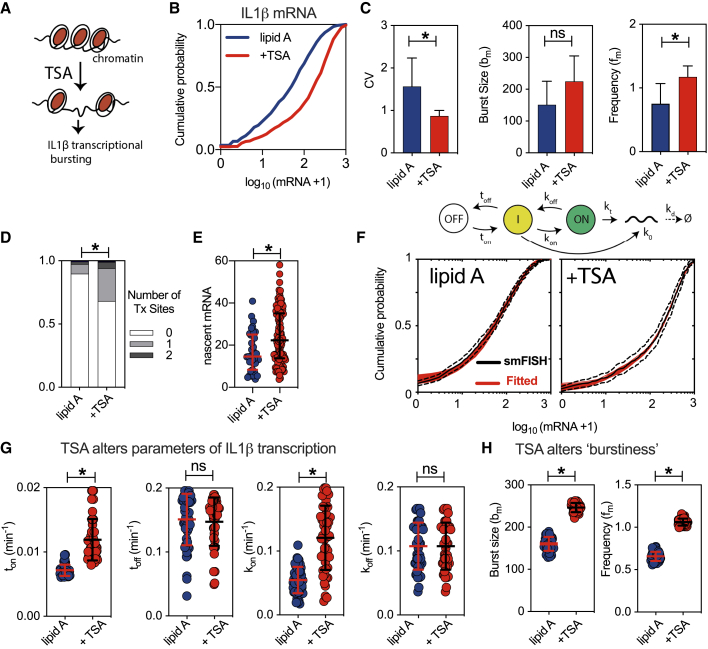
Modulation of Transcriptional Bursting via Chromatin State (A) Schematic representation of the treatment protocol: cells exposed to 10 μM TSA for 1 h before 500 ng/mL lipid A treatment. (B) TSA alters IL-1β mRNA distribution. Cumulative probability distribution of smFISH mRNA counts in BMDMs pre-treated with TSA prior to lipid A stimulation (+TSA; as in A), or control cells stimulated with lipid A. Shown is the *IL-1β* levels expressed as log_10_(mRNA+1) pooled across at least three replicates, from 732 (lipid A) and 305 (lipid A +TSA) cells, respectively. (C) Characteristics of single-cell mRNA expression. Shown is the CV, *b*_*m*_, and *f*_*m*_ calculated based on moments of the mRNA count data from (A) (expressed as mean ± SD from experimental replicates). “^∗^” denotes a result of a two-sample Mann-Whitney U test between groups (p < 0.05; ns, not significant). (D) Distribution of Tx in data from (B). Shown is the fraction of cells with 0–2 Tx. “^∗^” denotes a result of the Fisher exact test (p < 0.05) for the difference in the Tx site distribution. (E) Nascent IL-1β mRNA counts (with means and SDs) from 35 (lipid A) and 114 (lipid A +TSA) Tx from (D), respectively. “^∗^” denotes a result of two-sample Mann-Whitney U test between groups (p < 0.05). (F) Comparison between the measured and fitted *IL-1β* mRNA counts across conditions from (B). In black: Kaplan-Meier estimator of the measured CDF (with 95% confidence intervals); and in red: a family of models (50) fitted to the data. (Top) schematics of the fitted transcriptional model. (G) TSA modulates kinetic parameter rates in the fitted *IL-1β* models. Shown are selected parameter values (with mean and SD) for families of fitted models from (F). “^∗^” denotes a result of a two-sample Mann-Whitney U test between groups (p < 0.0001, ns). (H) TSA alters bursting characteristics of *IL-1β* expression. Shown are the moment estimates (mean and SD) of the burst size and frequency for fitted mRNA distributions from F. “^∗^” denotes a result of a two-sample Mann-Whitney U test between groups (p < 0.0001).

The observed temporal and spatial coordination of *IL-1β* and *IL-1α* expression is suggestive of epigenetic mechanisms. A transcriptional activator trichostatin A (TSA) was applied to selectively inhibit the class I and II histone deacetylase (HDAC) enzymes responsible for genome-wide chromatin accessibility (Figure 5A) (Vanhaecke et al., 2004). BMDMs pre-treated with TSA for 1 h prior to 3 h lipid A stimulation exhibited elevated *IL-1β* expression, notably, the expression of *TNF-α* was completely abolished (Figure 5B). The resulting *IL-1β* mRNA distribution was shifted toward higher mRNA counts (in comparison to the lipid A control) (Figure S22 for the lipid A dose response). TSA pretreatment significantly reduced the noise of *IL-1β* expression and altered burstiness by significantly increasing the moment estimate of bursting frequency, there was also an indication of changes in the burst size (Figure 5C). The number of active Tx sites was increased, consistent with more frequent activation of transcription following TSA treatment (Figure 5D). In comparison with the lipid A treatment, each *IL-1β* Tx site was also associated with more nascent mRNA (Figure 5E) indicative of a larger burst size. The differences in bursting characteristics were maintained across different lipid A doses (when co-treated with TSA), while the corresponding mean-variance relationships could not be statistically distinguished (Figure S22G). To quantitatively understand these mRNA expression patterns, mathematical modeling was applied (Figure S23). Consistent with the previous analyses, a two-step model was required to fit *IL-1β* mRNA distributions in both the control (untreated with TSA) and TSA pre-treated cells (Figure 5F). In comparison to the lipid A control, the TSA pretreatment was associated with quantitative changes in kinetic parameter rates consistent with chromatin regulation (Figures 5G and S23B). In the first permissive step, the gene switching “on” rate was increased (from 0.007 to 0.012 min^−1^, equivalent of a change in “off” time, from 142 to 83 min, for lipid A control versus lipid A +TSA, respectively) consistent with more frequent activation. Similarly, in the second step, TSA treatment also resulted in the increased “on” rate (from 0.05 to 0.1 min^−1^, the equivalent of a change in “off” time from 20 to 10 min). No further changes were observed in other model parameters, although the transcription rate corresponding to the permissive step was reduced following TSA treatment (Figure S23B). Overall, these changes resulted in a significant quantitative increase in the moment estimates of the burst size and burst frequency following TSA treatment (Figure 5H). Overall, these suggest that regulation of the chromatin state may allow concurrent regulation of the burst size and frequency and thus modulation of the *IL-1β* gene-expression output .

## Discussion

While single-cell responses exhibit substantial cell-to-cell variability, a fundamental question remains how this variability is constrained. Here, we considered an endogenous TLR system with known differential responses to a range of immune-relevant conditions (Adamik et al., 2013; Escoubet-Lozach et al., 2011; Hao and Baltimore, 2009; Meissner et al., 2013; Ramirez-Carrozzi et al., 2009; Tong et al., 2016). Using our quantitative smFISH data as well as published scRNA-seq data (Shalek et al., 2014), we showed that variability in the expression of TLR-induced genes is constrained by gene-specific trends over a large range of mRNA expression (Figure 3). This demonstrates that the stimulation (or perturbation) merely modulates the variability of mRNA expression as a linear function of its mean. We further predicted that this theoretically imposes the constraints on the underlying transcriptional bursting characteristics in response to stimulation (Figure 4). We also demonstrated that in the case of bursty mRNA production, the expression variability (in terms of the mean-variance relationship) is essentially defined by the burst size, while responses to environmental changes are controlled via frequency modulation. In general, we predicted that both burst size and frequency may undergo modulation, with the contribution of the former increasing as the system departures from the bursty mRNA production regime. We validated these predictions using our *TNF-α* and *IL-1β* smFISH measurements as well as provided supporting evidence in a large set of TLR-regulated genes in primary immune cells (Figure 4). Finally, we demonstrated that modulation of chromatin state may account at least, in part, for the predicted modulation of transcriptional bursting of *IL-1β* expression (Figure 5).

We hypothesized that the observation that empirical mean-variance relationships are gene-specific, linear, and maintained over different conditions represent a fundamental property of a gene regulatory system. Our results imply an inverse relationship between the mean expression level and noise (Dar et al., 2016), while providing insight into the regulation of gene expression in response to stimulation. In agreement with previous work, we found that higher expression is accompanied by increased burstiness (Sanchez and Golding, 2013), while different levels of noise are associated with distinct subsets of parameter values (Figure S14). While not all the possible combinations of rates are ever explored in a biological system (Hausser et al., 2019), the fitted parameter changes (along with mean-variance trends) are dominated by modulation of gene activity switching rates, while modulation of transcription rates are associated with low transcriptional output (Figures S16 and S17). Our results perhaps reflect a related set of immune conditions used in the study (i.e., related ligands, dose-responses, co-stimulation, or generic chromatin perturbation), essentially affecting a large, well-connected signaling network (Oda and Kitano, 2006). As such, we found that these signals and conditions only modulate kinetic parameters of *TNF-α* and *IL-1β* transcription, rather than induce substantial changes in the mode of regulation. Recent analyses suggest that burst sizes are encoded within core promoters (Larsson et al., 2019). In fact, promoters of highly variable cytokine and chemokine genes are enriched for TATA boxes (Figure S3) and are depleted of CpG islands, in comparison to low heterogeneity TLR-dependent genes (Hagai et al., 2018). In turn, the frequency may be modulated via histone acetylation (Nicolas et al., 2018) or TF signaling events (Hagai et al., 2018; Wong et al., 2018). In the TLR system, the latter is likely related to the levels of upstream TFs or their activation patterns. For example, although heterogeneous, the NF-κB system activation exhibits dose-dependent and temporal regulation (Adamson et al., 2016; Bagnall et al., 2018; Muldoon et al., 2020; Selimkhanov et al., 2014; Sung et al., 2014; Wong et al., 2019), which might enable the fine-tuning of the underlying transcription and gene activation rates. It would be intriguing to understand whether gene-specific trends are sensitive to therapeutic compounds, or, in fact, pathogen stimulation. It would also be relevant to understand the modulation of parameter changes more broadly, i.e., in transcriptomics data, which in this work we only analyzed in terms of relative burstiness (Figure 4F). In particular, the linear mean-variance relationships theoretically imply a generic reciprocal relationship between the burst size and frequency (Figure 4), which would require further validation using more gene targets. While computationally feasible (Larsson et al., 2019), the present scRNA-seq dataset (Shalek et al., 2014) does not provide quantification of mRNA numbers, which is required to fit models accurately.

As a key part of this study, we quantitatively characterized the regulation of the TNF-α and IL-1 cytokines, which encode distinct roles during inflammatory responses and pathogen recognition (Lu et al., 2015). The expression of the short-lived *TNF-α* mRNA transcript conforms to a simple one-step stochastic model of transcription (Raj et al., 2006; Skinner et al., 2016; Suter et al., 2011; Zenklusen et al., 2008), consistent with frequent transcription initiation but limited transcriptional output per burst (Figures 1 and 2). The transcription of *IL-1β* is characterized by lower bursting frequency and larger burst sizes compared with that of *TNF-α*. This behavior does not conform to a one-step model, and through mathematical modeling we showed that an intermediate regulatory step is required to explain the observed mRNA distributions. While previously considered complex models involved a promoter cycling step (Harper et al., 2011; Zoller et al., 2015), in our model, the intermediate step is associated with a low transcriptional output. This model structure is supported by the experimental evidence for a permissive step due to chromatin regulation. First, we observed biphasic patterns of *IL1α* and *IL1β* mRNA synthesis (Figure S21, which is also evident in previously published data; Shalek et al., 2014; Figure S5). Second, we found a marked temporal and spatial correlation between on-going *IL-1β* and *IL1α* transcription (as indicated by the co-localization of active transcription sites observed with smFISH; Figure S21), which likely underlies an association in the local chromatin structure (Iborra et al., 1998). Third, the chromatin modulator TSA altered the *IL-1β* mRNA distribution, resulting in more frequent mRNA bursts consistent with the two-step model (Figure 5). While elevating *IL-1β* expression, TSA treatment completely ablated the expression of *TNF-α* mRNA. This suggests that chromatin regulation may enable cytokine-specific control of the effector responses. In general, the co-association of multiple genes within common centers of mRNA synthesis provides an additional layer of regulation for gene expression, in which the combination of genes within an active factory might contribute synergistically to the timing, duration, and extent of synthesis from the spatially co-associated genes (Fanucchi et al., 2013; Li et al., 2012; Schoenfelder et al., 2010). The specific mechanisms involved in the regulation of the permissive chromatin states and robust *IL-1β* expression are not fully understood, but both cell-specific (e.g., heterogeneous signaling events) or cell-extrinsic (e.g., paracrine signaling) processes affecting TF-activation patterns (Lu et al., 2015; Shalek et al., 2013, 2014; Xue et al., 2015) might contribute to this. How many genes share complex modes of regulation, or, in general, whether functionally related genes exhibit co-variability in their responses is unclear. *IL1α* and *IL1β* represent one example of co-variability. It is currently unknown whether the heterogeneity of the *IL1β* gene and protein expression described here is fundamentally linked to the apparently cell-specific inflammasome activation as a mechanism to control cytokine levels in circulation and to minimize inflammasome-mediated cell death (Daniels et al., 2016). Further studies will also be required to quantitatively link the underlying TF dynamics, epigenetic control, and the target gene transcription, as well as protein expression and secretion (Junkin et al., 2016; Lee et al., 2014; Singer et al., 2014).

In summary, the study demonstrates that despite seemingly noisy responses, the heterogeneity of the single-cell and population-level TLR effector responses is defined by fundamental functional constraints. We propose that the constrained variability of the TLR-dependent gene response might be a key element of the antibacterial and inflammatory response and may constitute a common feature of inducible gene-expression systems in general.

## STAR★Methods

### Key Resources Table

**Table:** 

REAGENT or RESOURCE	SOURCE	IDENTIFIER
**Antibodies**		

Anti-IL1β	Abcam	Cat#Ab9722; RRID AB_308765
Anti-Rabbit (Alexa Fluor® 488 conjugated)	Abcam	Cat#Ab150077; RRID AB_2630356

**Chemicals, Peptides, and Recombinant Proteins**		

Lipid A Salmonella Minnesota Re595	VWR	Cat#CA80056-964
Recombinant mouse interferon-γ	Thermo Fisher	Cat#PMC4031
Dimethyloxalylglycine	Sigma-Aldrich	Cat#D3695
Trichostatin A	Sigma-Aldrich	Cat#T8552
Vectashield (with DAPI)	Vector Laboratories	Cat#H-1200

**Critical Commercial Assays**		

C1 Single-Cell Auto Prep IFC for mRNA Seq (10 – 17 mm)	Fluidigm	Cat#100-6041; RRID:IMSR JAX:000664
SMARTer Ultra Low RNA Kit for the Fluidigm C1 System	Clontech Takara	Cat#P/N 634833
Nextera XT DNA library preparation index kit	Illumina	Cat#FC-131-1002

**Deposited Data**		

scRNA-seq of RAW 264.7 cells to lipid A	This paper	https://www.ebi.ac.uk/arrayexpress/experiments/E-MTAB-9219/
Codes for fitting mathematical models and scRNA-seq analyses, parameters of fitted models	This paper	https://github.com/ppaszek/transcriptionalBursting
scRNA-seq analysis of BMDC cells	(Shalek et al., 2014)	https://www.ncbi.nlm.nih.gov/geo/query/acc.cgi?acc=GSE48968
Mouse reference genome NCBI build 38, GRCm38.p3	Genome Reference Consortium	http://www.ncbi.nlm.nih.gov/projects/genome/assembly/grc/mouse/
Eukaryotic Promoter Database	EPD	https://epd.epfl.ch//index.php

**Experimental Models: Cell Lines**		

RAW264.7	ATCC	TIB-71; RRID CVCL_0493

**Experimental Models: Organisms/Strains**		

C57/BL (BMDM preparation)	Jackson Laboratory	JAX: 000664; RRID:IMSR JAX:000664

**Oligonucleotides**		

Probe sequence for smFISH (Table S2)	This paper	N/A

**Software and Algorithms**		

MATLAB	MathWorks	RRID:SCR_001622
FISH-QUANT	(Mueller et al., 2013)	https://www.bitbucket.org/muellerflorian/fish_quant/src/master/
GraphPad Prism 8	Graphpad software	RRID:SCR_002798
Cell Tracker	CellTracker software	https://www.warwick.ac.uk/fac/sci/dcs/people/till_bretschneider/celltracker/
Casava 1.8.3	Illumina	http://support.illumina.com/sequencing/sequencing_software/casava.html
Tophat 2.011	(Trapnell et al., 2009)	RRID:SCR_013035
Picard Tools	Piccard Tools software	RRID:SCR_006525
Matrix exponent algorithm	(Al-Mohy and Higham, 2011)	https://github.com/higham/expmv
SoftWoRx 7.0	GE Healthcare	https://cdn.gelifesciences.com/dmm3bwsv3/AssetStream.aspx?mediaformatid=10061&destinationid=10016&assetid=17238
Stellaris RNA FISH Probe Designer	LGC Biosearch Technologies	https://www.biosearchtech.com/support/tools/design-software/stellaris-probe-designer

### Resource Availability

#### Lead Contact

Further information and requests for resources and reagents should be directed to and will be fulfilled by the Lead Contact, Pawel Paszek (pawel.paszek@manchester.ac.uk )

#### Material Availability

This study did not generate new materials

#### Data and Code Availability

The sequencing data generated during this study are available at ArrayExpress under accession no E-MTAB-9219 (https://www.ebi.ac.uk/arrayexpress/experiments/E-MTAB-9219/). MATLAB and Python codes generated during this study are available via Github [https://github.com/ppaszek/transcriptionalBursting]. The raw smFISH data including all the image files are too large to upload to existing public repositories, but these are available upon request.

### Experimental Model and Subject Details

#### Cell Culture

RAW 264.7 male murine macrophages (obtained from ATCC) were cultured in Dulbecco’s modified eagle medium supplemented with 10% foetal bovine serum (Gibco) and 1% non-essential amino acids as described previously (Bagnall et al., 2015). Cells were not authenticated. Primary BMDMs were differentiated from bone marrow taken from the hind legs of adult 8-12 weeks male or female C57BL/6 mice (not involved in other procedures). Isolated cells were disrupted and homogenized by repeating pipetting until no lumps were visible. The cell suspension was then centrifuged at 200 g for 5 min and the resulting pellet re-suspended in DMEM (supplemented with 100 units/ml penicillin, 100 ug/ml streptomycin (all from Sigma-Aldrich, UK), 10% FCS (Gibco, UK), and 30% L929 cell-conditioned media) and then plated. After 72 h the media were replaced with fresh supplemented media. Cells were harvested (by washing with cold PBS) on day 6-8 and used for experiments within 24 h.

#### Reagents

Cells were stimulated with various doses of lipid A Salmonella Minnesota Re595 (VWR), 100ng/ml recombinant mouse interferon-γ (Life Technologies), 0.5mM dimethyloxalylglycine (Sigma-Aldrich) or 10μM Trichostatin A (Sigma-Aldrich). Slide mounting and nuclei staining was performed using Vectashield mounting medium with DAPI (Vector Laboratories).

### Method Details

#### Single-Cell RNA-seq

Single-cell sample collection and preparation was performed using the C1 Fluidigm platform, using the manufacturer’s instruction. A suspension of appropriately stimulated 1x10^6^ RAW 264.7 cells per ml was prepared in serum-free media and appropriately mixed with C1 suspension reagent. The resulting cell mixture was then loaded into C1 Single Cell AutoPrep IFC microfluidic chip (calibrated for medium 10-17μm cell sizes). The microfluidic chip was then placed into the C1 Fluidigm system for processing, using the ‘mRNA Seq: Cell Load’ script. Verification of single-cell capture was performed by wide-field microscopy. Single-cell library construction was performed using the SMARTer Ultra Low RNA reagent kit (Takara®) for cDNA amplification, followed by the Nextera® XT DNA Index kit for fragmentation and barcoding of samples (Illumina®). DNA sequencing was performed by paired-end sequencing (100 + 100 cycles, plus indices) on an Illumina HiSeq2500 instrument.

#### Single-Molecule RNA-FISH

Custom Stellaris® FISH probes were designed against murine *IL1α* (NM_010554), *IL1β* (NM_008361) and *TNFα-* (NM_013693) cDNA sequences by utilising the Stellaris® FISH Probe Designer (Biosearch Technologies, Inc., Petaluma, CA). *IL1α* probes were conjugated with the Quasar-570 dye. *TNFα* and *IL1β* probes were conjugated with either the Quasar-570 dye or Quasar-670 dye for multiplexing with *IL1α* probes (see Table S2 for tabulated smFISH counts and probe lists). Cells were plated into 12-well plates containing sterilised glass cover slips. After adherence, appropriately stimulated cells were fixed and labelled using Stellaris® protocol, following manufacturer’s instructions (including co-immunofluorescence for protein levels). Samples were imaged using a DeltaVision (Applied Precision) wide-field microscope with a 60x/N.A.1.42 oil immersion Plan Apo N objective and Sedat Quad filter set was used. The images were collected using a Coolsnap HQ (Photometrics) camera with a z optical spacing of 0.2 μm.

#### Immunofluorescence

Cells were plated onto sterile glass coverslips submerged in media, and after adherence stimulated as required. Cells were fixed by immersion in 4% paraformaldehyde for 15 mins and then washed with PBS. Samples were incubated in the presence of 1:100 anti-IL1β primary antibody (abcam; ab9722) for 1 h at room temperature, washed and further incubated for 30 mins in the presence of 1:500 secondary antibody (abcam; ab150077) before a final PBS wash. The glass coverslip was then mounted on to a glass slide ready for imaging. Confocal microscopy was used to visualise anti-IL1β staining. FITC conjugates were excited using a 488 nm laser line and emitted signal detected after passing through a 505-550 nm bandpass filter, using LSM510 photomultiplier detectors. For quantitative comparison of fluorescence, all images were taken together using the same detector settings. Fluorescence levels were quantified using Cell Tracker Version 0.6 (Shen et al., 2006).

#### Experimental Design

smFISH data are representative of at least 2 biological replicates, scRNA-seq analysis of lipid A stimulated RAW 264.7 cells were performed using 1 replicate. Data were not randomized, stratified, or blinded for any of the analyses performed in this paper.

#### Stochastic Modelling of Transcription

##### CME Description

Temporal mRNA distributions for considered models of transcription are obtained using the Chemical Master Equation (CME) following approach by (Gómez-Schiavon et al., 2017). In brief, an infinite set of ordinary differential equations (ODEs) describes the flow of the probability in the biochemical system being in a particular state *x* and time *t*, *P(x,t)* over all possible biochemical reactions *k* into and out of *x*:

$$\frac{dP\left(x,t\right)}{dt}=\sum_{k}\left[a_{k}\left(x-v_{k}\right)P\left(x-v_{k},t\right)-a_{k}\left(x\right)P\left(x,t\right)\right]$$

$a_{k}\partial t$ denotes the probability that a biochemical reaction *k* will occur in the infinitesimal time interval ∂t, given that the system is in the state *x*, $v_{k}$ is a stochiometric vector of reaction *k* that describes how the system changes when reaction *k* occurs. In general, CME is written in the matrix form as

$$\frac{dP\left(X,t\right)}{dt}=R\left(\theta \right)P\left(X,t\right)\text{,}$$

where $X={\left[x_{1},x_{2},\ldots x_{N}\right]}^{T}$ is a vector of all possible cell states, $P\left(X,t\right)={\left[P\left(x_{1},t\right),P\left(x_{2},t\right),\ldots P\left(x_{N},t\right)\right]}^{T}$ and R(θ) is a transition rate matrix given by:

$$R_{ij}\left(\theta \right)=\left\{ \begin{array}{c} -\sum_{k}a_{k}\left(x_{i}\right)\;if\;i=j\;\\ a_{k}\left(x_{j}\right)\;\forall j\;\text{such}\;\text{that}\;x_{j}=x_{i}-v_{k}\\ 0\;\text{otherwise.}\; \end{array}\right.$$

Time evolution of the probability distribution P(X,t) is given by

$$P\left(X,t\right)=\exp\left[R\left(\theta \right)t\right]P_{0}\left(X\right)\text{,}$$

where $P_{0}\left(X\right)$ is specified by initial data that should satisfy $\sum_{X}P_{0}\left(X\right)=1$. P(X,t) is calculated using a fast matrix exponential function implemented in MATLAB by (Al-Mohy and Higham, 2011). All simulations begin with initial data in which no mRNA are present and both gene alleles in the ‘*off*’ state. For practical purposes, the total number of mRNA molecules in the system—and hence the total number of states in the stochastic process—is truncated at *M =2000*.

In general, R(θ) depends on both the model structure and the parameters. In this work, we considered a family of four transcriptional models of increasing complexity (as highlighted in Figure S11D). In the simplest model—often called the *telegraph model*—we assume two independent alleles for each gene, the activity of which switches randomly between ‘*off*’ and ‘*on*’ states, with only the latter being permissive for mRNA transcription (Raj et al., 2006; Skinner et al., 2016; Suter et al., 2011; Zenklusen et al., 2008). The associated kinetic parameters include switching ‘*on*’ and ‘*off*’ rates (*k*_*on*_ and *k*_*off*_, respectively) as well as rates of mRNA transcription and degradation (*k*_*t*_ and *k*_*d*_, respectively). The state of the cell in the telegraph model x *∈ [s, m]*^*T*^ is defined by the number of active alleles, *s* and number of mRNA molecules, *m*. The total number of states is *N = 3(M +1)*, subject to the constrains on the number of mRNA molecules *M*. A considered variant of the model includes an additional constitutive transcription rate *k*_*0*_, which is incorporated into the transition matrix (see Figure S11D model 2).

We also consider an extension to the telegraph model that includes an additional regulatory step, which may be considered as a chromatin opening step that is required for full transcriptional activity. In the extended models, each allele exists in one of three states: an inactive ‘*off*’ state, an intermediate ‘I’ state or an active ‘*on*’ state. Reversible stochastic transitions (with appropriate rates) occur between the inactive and intermediate as well as the intermediate and active states (but not directly between inactive and active states). We further assume that transcription occurs only in the active state (Figure S11D model 3) or in both the intermediate and active states (i.e. *IL1β* model, Figure S11D model 4). Given the upper bound on the number of mRNA molecules *M*, the total number of states in the extended models is *N = 6(M +1)*.

#### Model Fitting and Analysis

In order to investigate different regulatory scenarios (Figure S11B), we calculated exact temporal mRNA distributions using the CME approach as sketched above. A genetic algorithm (GA) was implemented using the *ga* function in MATLAB and employed to estimate model parameters, minimising the integrated absolute distance between the theoretical (CME) and measured cumulative distribution functions (CDFs). CDFs were fitted using *fitdist* function (with an Epanechnikov kernel function). The best 50 model fits from independent GA runs for each condition (using a population size of 200, elite count of 2, crossover factor of 0.6, and the tournament selection function). Gene activation rates were constrained to lie below 0.2 min^-1^, while the degradation rate for *TNFα* transcripts was constrained to lie between 0.006 and 0.07 min (half-life between 10 and 115 mins), while the degradation rate for *IL1β* transcripts was constrained to lie between 0.002 and 0.006 min (half-life between 115 and ~350 mins). This is in an agreement with a short *TNFα* mRNA half-life (up to 1.5 h) in comparison to that of *IL1β* (stable at the time-scale of a 6 h experiment) measured in macrophages (Hao and Baltimore, 2009). We assumed two independent alleles per gene with the transcription rate constrained by 30 mRNA min^-1^ per allele. Rates as high as 2 to 10 mRNA min^-1^ have been reported for specific genes (Molina et al., 2013; Schwanhäusser et al., 2011; Skinner et al., 2016; Suter et al., 2011). In our dataset 10% of RAW 264.7 cells produced in excess of 200 mRNA h^-1^ (and 1% in excess of 400 mRNA h^-1^), which is equivalent to a transcription rate between 1.67 to 3.33 mRNA min^-1^ per allele assuming constant production and no decay. Note that these are underestimates, as they assume steady production, while our transcription site data indicates intermittent transcriptional initiation.

The CME approach was also used to calculate sensitivity indexes corresponding to 10% parameter changes of the noise level [1-σ_10_/μ_10_/(σ_0_/μ_0_)], where σ_0_ and μ_0_ correspond to nominal parameter values. Sensitivity indexes were calculated for distributions obtained at 180 mins after stimulation for one-step model for TNFα (Figure 2D), two-step *IL1β* model (Figure 2E) or one-step model refitted to recapitulate heterogeneity of *IL1β* expression (Figure S11F).

#### Noise Quantification in Count Data

Single-cell heterogeneity may emerge due to intrinsic stochastic fluctuations (i.e., random on-off switching) and extrinsic differences between cells (Elowitz et al., 2002; Hilfinger and Paulsson, 2011; Sherman et al., 2015). Therefore, in order to apply stochastic models of transcription (which assume intrinsic noise), we investigated the sources of the variability in the smFISH count data. Overall, these analyses suggest that intrinsic noise is a dominant factor in our datasets. In agreement, we show that one-step telegraph models explain all, but ~ 30% variability in data for *TNFα* smFISH counts (Figure 4B), while two-step model capture most of the variability in the *IL1β* data (Figure S18C).

(1) We demonstrate that count data exhibit intrinsic noise properties:
  (i) Noise decreases monotonically with mean μ in smFISH data (Figure S11A) as well as in our (Figure S3C) and published scRNA-seq (Figure S20E).
  (ii) In the limit of high μ, noise decreases sharply (Figure S11A), rather than approaching a plateau (Taniguchi et al., 2010).
  (iii) *TNFα* smFISH counts (Figure S16) as well as a majority of the scRNA-seq distributions (Figure S20A) fit negative binomial distributions. Of note, *IL1β* smFISH counts do not follow negative binomial distribution (Figure S16), since they represent a more complex model of regulation.
(2) A formal noise decomposition of the *TNFα* and *IL1β* dose-response count data (Rhee et al., 2014) shows that contribution of the intrinsic noise is dominant (across most condition), albeit also highlighted an extrinsic noise component (Figure S11B). To analyse potential sources of extrinsic noise, we show that
  (i) The percentage of variance of *TNFα* and *IL1β* smFISH counts explained by cell size (R^2^ of the linear fit) is <7% (Figure S10B).
  (ii) The percentage of variance explained by regressing *TNFα* against *IL1β* counts is <20%, but ~41% for *IL1α* against *IL1β* (Figure S21). The former is consistent with extrinsic variability due to shared TLR signalling machinery, for example signalling dynamics (Wong et al., 2018, 2019), while the latter highlight the shared chromatin regulatory step.
(3) Currently, our smFISH datasets include between 10^2^ and 10^3^ individual cells per conditions (up to 18 conditions per probe) and up to 96 cells in scRNA-seq. In general, larger sample sizes might allow obtaining more accurate estimates of the underlying probability distribution function and their moments.

### Quantification and Statistical Analysis

#### Analysis of scRNA-seq Data

Demultiplexing of the output data (allowing one mismatch) and BCL-to-Fastq conversion was performed with CASAVA 1.8.3. Reads were mapped to the *mus musculus* genome (assembly GRCm38.p3, downloaded from Ensembl) using Tophat version 2.011 (Trapnell et al., 2009) and assigned to genomic features in the corresponding gtf file using featureCounts in the Subread package (version 1-4.6). Counts for each gene were normalized to the median counts per cell (Figures S1A and S1B), data is presented as *log*_*2*_*(normalised counts+1)* following analyses by (Shalek et al., 2013). 61 cells were included in the analysis with more than 2 million counts. A PCA plot of the normalized counts reveals a relatively uniform distribution of cells with no outliers or apparent overriding trends in the projection (Figure S1C). Comparison of normalized gene expression counts between two representative single cells shows a relatively strong correlation as observed in Figure S1D. Expression of housekeeping genes displays an almost linear correlation between these two cells (Spearman rank correlation of *0.94*). In contrast, genes whose expression was regulated in response to lipid A, display far greater variation (Spearman rank correlation of *0.71*). The mean of the transcript levels from the single cells were then compared to previously published population-level data performed under the same experimental conditions (Bagnall et al., 2015). These data were downloaded, re-mapped and re-normalised (as described here) to ensure parity between the datasets. A strong correlation between the population-level and single-cell data (*r=0.85*) was observed, confirming that library preparation preserved overall gene expression patterns (Figure S1E). As in Shalek et al (Shalek et al., 2013) we analysed the correlation between mean normalized counts (across all 61 cells) and the variability of these counts. We observe an inverse relationship between the normalized mean counts and the coefficient of variation (Figure S1F). All the housekeeping genes exhibit extremely low variability across the cells, while the lipid A responsive genes show far greater variability at comparable expression levels. Subsequently, a stringent cut-off was enforced to remove genes with high technical variability, leaving 1941 high-confidence genes. Data was clustered using the affinity propagation algorithm (Frey and Dueck, 2007); an unsupervised non-parametric method, which provides automated determination of numbers of clusters. Derived p-values were corrected for multiple testing using the method described by Benjamini and Hochberg (see Table S1 for normalised read counts and clustering analyses).

#### Generic Properties of the TLR4 Response

Eukaryotic promotor database (EPD) was used to determine TATA-box enrichment in the clusters displayed in Figure 1B. We observe significant enrichment of TATA sites in the promoter regions of the highly variable genes that failed to cluster (8 out of 10 genes have TATA boxes in their promoter regions, Figure S3A). In contrast, we do not find enrichment for TATA boxes in the promotor regions of the housekeeping genes examined. When comparing the variability in transcript levels of all genes within the (HC) single-cell dataset with and without TATA boxes, we find there is no statistical difference between the two sets (Figure S3B). In part this may be determined by the cut-off in transcript levels of high confidence genes, i.e. the HC is by definition less variable. Previously correlation has been found between variability in transcript level and mRNA half-life in a single cell study examining a variety of rat and mouse tissues (Dueck et al., 2015). A plot of the variability in expression levels of genes within this dataset and previously published mRNA half-lives (Schwanhäusser et al., 2011) reveals a limited negative correlation between half-life and heterogeneity, perhaps due to the fact that all recorded half-lives are large (e.g., >4 h, Figure S3B). Yet, there is a strong association between mRNA abundance (and transcript synthesis rate) and variation, indicative of a generic relationship between abundance and noise.

#### smFISH Quantification

Raw images were deconvolved using the SoftWoRx 7.0 software (GE Healthcare). Spot counting for mature and nascent mRNA was performed with FISH-Quant v2d (Mueller et al., 2013). The total cell area was calculated by extracting the number of pixels and pixel size in each drawn cell boundary. The nuclear area was calculated by applying the MATLAB function ‘greythresh’ to the maximum projection of the deconvolved DAPI signal. Pixel areas for each nuclear mask were extracted and scaled to the actual pixel sizes. For cell size normalisation, each individual mRNA count was scaled via the ratio of the average nuclear area of the population and nuclear area of the cell (Figure S10).

#### Point Estimators of Transcriptional Bursting

Transcriptional burst size and burst frequency are defined as the average number of mRNA produced per gene activation event, and the frequency of gene activation events, respectively (Nicolas et al., 2017). In the case of the one-step telegraph model, these are directly related to the kinetic parameters of transcription (Nicolas et al., 2018). When accounting for two independent alleles of a gene, in the steady-state burst size is defined as *b*_*k=*_*k*_*t*_*/k*_*off*_, while bursting frequency is given by *f*_*k*_*=2k*_*on*_*k*_*off*_*/(k*_*on+*_*k*_*off*_*)*/*k*_*d*_ (we refer to these as *kinetic estimators*). Alternatively, estimators based on the sample variance *σ*^2^ and the mean *μ* of the mRNA distribution (referred to here as *moment estimators*) such that the burst size (*b*_*m*_*=σ*^*2*^*/μ*) and burst frequency [*f*_*m*_*=μ/(b*_*m*_*-1)*] are sometimes used (Nicolas et al., 2017; Raj et al., 2006; Suter et al., 2011; Wong et al., 2018). In general, moment estimators are used to describe burstiness, i.e. quantitative departure from a *non-bursty* (Poissonian) mRNA production (where *b*_*m*_=*1 and f*_*m*_*=∞*) (Nicolas et al., 2017; So et al., 2011; Wong et al., 2018). To evaluate the difference between estimators we define an error function:

$$Error=\;\frac{kinetic\;parameters\;estimate-moment\;estimate}{kinetic\;parameters\;estimate}$$

Given expressions for the steady-state mRNA moments in the telegraph model (Peccoud and Ycart, 1995; Paszek, 2007; Shahrezaei and Swain, 2008), when accounting for two alleles we have that

(Equation 1) $$\text{μ}=\frac{2k_{on}k_{t}}{k_{d}\left(k_{off}+k_{on}\right)}$$

(Equation 2) $${\text{σ}}^{2}={\frac{\text{μ}}{2}}^{2}\frac{k_{off}}{k_{on}\left(1+\;\frac{\left(k_{off}+k_{on}\right)}{k_{d}}\right)}$$

In this work, we use moment estimators calculated either for smFISH and scRNA-seq data or for theoretical mRNA distributions (at any arbitrary time) obtained from the CME. For application of the one-step telegraph model we utilise kinetic parameter estimators.

Therefore, errors in the steady-state may be expressed as

(Equation 3) $$b_{error}=1-\;\frac{{k_{off}}^{2}}{k_{d}\left(k_{off}+k_{on}\right)+{\left(k_{off}+k_{on}\right)}^{2}}-\frac{k_{off}}{k_{t}}\text{,}$$

(Equation 4) $$f_{error}=1-\frac{\left(k_{off}+k_{on}+k_{d}\right)\left(k_{off}+k_{on}\right)}{{k_{off}}^{2}}\text{.}$$

In general, the error associated with moment estimators depends on specific parameter values and the error in the bursting frequency is independent from the transcription rate *k*_*t*_. As already well established in the literature (Nicolas et al., 2017), in the ‘bursting’ regime, corresponding to short and infrequent activation events, i.e. *k*_*off*_*≫k*_*on*_ and *k*_*d*_*≫k*_*off*,_ errors resulting from moment estimators are negligible (given that in general *k*_*t*_*≫k*_*off*_), Equations 3 and 4. In this case both errors converge to 0, and thus moment estimators are as accurate as kinetic parameter estimators. In order to understand the generic suitability of moment estimators, we calculated errors associated across a wide range of parameter values using the fitted TNFα model (Figure S6). In the physiological parameter range, i.e. *k*_*on*_*<0.1 min*^*−1*^ and *k*_*off*_ *<0.2 min*^*-1*^, and assuming *k*_*t*_*<30 mRNA/min*^*-1*^, both errors are constrained (*f*_*error*_*<1* and *b*_*error*_*<1*) for *k*_*off*_*>k*_*on*_ (Figure S6A). In the case of TNFα, where *k*_*off*_*/k*_*on*_ is equal to 6, the errors due to approximation via moment estimators are ~30% (see also Figure S11G). These errors substantially increase when *k*_*off*_*/k*_*on*_ *~1*, but are independent of transcription rate (at least above 5 mRNA/min, Figure S6B). Both errors also depend on the mRNA half-life, but within the physiological range, i.e. *k*_*d*_*<0.01 min*^*-1*^ the corresponding changes are limited (for *k*_*off*_*/k*_*on*_ *>3*). While kinetic estimators define bursting characteristics only at the steady-state, the moment estimators can be calculated at any time (Figure S6C). Temporal relationships (calculated based on theoretical distribution at 1, 3 and 6h) converge to the steady-state approximation (Figure S6A).

#### Modulation of Transcriptional Bursting

We theoretically calculated relationships between parameters of the telegraph model that satisfy empirically observed linear mean-variance relationships. We assume that the sample mean and variance of the gene expression distribution follows a general linear trend,

(Equation 5) $$\sigma^{2}=\alpha \mu +\sigma_{0}\text{.}$$

Under steady-state assumption, i.e. by using Equations 1 and 2, with *σ*_*0*_*=0*, this relationship corresponds to

(Equation 6) $$k_{t}=\left(\alpha -1\right)\left(k_{off}+k_{on}+k_{d}\right)\left(1+\frac{k_{on}}{k_{off}}\right)\text{,}$$

whereas in general (*α*_*0*_*≠0*):

(Equation 7) $$\frac{k_{off}k_{on}k_{t}^{2}}{k_{d}\left(k_{d}+k_{off}+k_{on}\right){\left(k_{off}+k_{on}\right)}^{2}}+\left(1-\alpha \right)\frac{k_{on}k_{t}}{k_{d}\left(k_{off}+k_{on}\right)}-\frac{\sigma_{0}}{2}=0\text{.}$$

The above equations define the relationship between kinetic parameters that satisfy linear constraints. Equation 6 describes a surface in the three-dimensional ($k_{off},k_{on},k_{t}$) parameter space on which the $\sigma^{2}=\alpha \mu$ relationship holds. We plot this surface, as well as bursting characteristics on the surface for *α=100* and *α=10* (corresponding to genes with different level of variability) for biologically plausible set of parameters, i.e. *k*_*off*_*<0.2 min*^*-1*^ and *k*_*on*_*<0.1 min*^*-1*^, while assuming *k*_*d*_ *=0.014 min*^*-1*^ (i.e., fitted *TNFα* degradation rate) and *k*_*t*_*<30 min*^*-1*^ (Figures S14A*–S14C*).

To maintain a linear mean-variance relationship the system can move freely between different ($k_{off},k_{on},k_{t}$) parameter values, which results in modulation of both burst size and frequency. In the case of bursty expression regime, i.e. *k*_*off*_≫*k*_*on*_ (for *k*_*off*_*≫k*_*d*_) it follows from Equation 6 that *b*_*k*_*=α-1* and *f*_*k*_*=μ/(α-1)*. Therefore, burst size is necessarily constant (and equal to the slope of the mean-variance line for large α) over the range of mean mRNA response, while changes of gene expression are controlled solely by frequency modulation. If the system is not in the bursty regime, the extent of burst size and frequency modulation is related to the activation rate *k*_*off*_ (or in general *k*_*off*_*/k*_*on*_ ratio, Figure S15). Based on the (*k*_*off*_*, k*_*on*_*, k*_*t*_) parameter surfaces, the relationship between bursting frequency and relative change of the burst size was calculated for a range of regression slopes from α=10 to 200, (Figures S15A and S15B). We find that the larger *k*_*off*_, the smaller are changes of the burst size, and in turn the larger are changes of the bursting frequency over the corresponding change of mean mRNA expression (Figure 4A).

When the system approaches a bursty regime, i.e. *k*_*off*_*≫k*_*on*_ the changes of burst size become negligible (Figure S15C). In this case, moment estimators provide an accurate description of the bursting characteristics for the one-step telegraph model (i.e., for α≫1 moment and kinetic estimators are the same).

The generic case of non-zero intercept, i.e. *σ*^*2*^*= αμ+σ*_*0*_ is considered in Equation 7, where parameter regions consist of two roots of the quadratic equation (see Figures S14D and *S14*E). One of these roots overlaps with the solution of Equation 6 (the case of non-zero intercept), while the second, associated with a small transcription rate (Figure S14E) disappears as *σ*_*0*_*→0*. In the bursty regime, Equation 7 can be re-arranged as

$$f_{k}b_{k}^{2}+\left(1-\alpha \right)fb-\sigma_{0}=0\text{.}$$

Given that *μ=ƒb*, we have that

(Equation 8) $$\begin{array}{c} b_{k}=\left(\alpha -1\right)+\frac{\sigma_{0}}{\mu }\\ f_{k}=\frac{\mu }{b_{k}}\text{.} \end{array}$$

Equation 8 show that for *σ*_*0*_*≠0* the burst size is a non-monotonic function of the mean μ that diverges as μ tends to zero. When μ≫0 the burst size tends asymptotically to the constant value $b_{k}=\left(\alpha -1\right)$, so that the description is equivalent to using moment estimators, in the sense that the burst size is predetermined by the slope of the mean-variance line. Of note, for *σ*_*0*_*>0* burst size relationship has a minimum for *μ*=*σ*_*0*_/*(α-1)*] and burst size monotonically increase (and *vice versa* for *σ*_*0*_*<0*, Figures 4C and 4D). In this case, the description is equivalent to using moment estimators, such that the burst size is predetermined by the slope of the mean-variance line, and constant in the case of the zero intercept, i.e. *b*_*m*_*=σ*^*2*^*/μ=α +σ*_*0*_*/μ, while* for α≫1, *b*_*m*_= *b*_*k.*_ In addition, the frequency undergoes modulation as a function of the mean, i.e. *f*_*m*_*=μ/(b*_*m*_*-1)=μ/[(α-1)+σ*_*0*_*/μ=f*_*k*_ *]*.

The comparison between moment (and kinetic estimators) is depicted in Figure 4C (in the case of a non-zero intercept for the fitted *TNF-α* smFISH data). The burst and frequency relationships are predicted based on coefficients of the linear mean-variance relationships (Figure 4B). In the case of *IL1β* (where the complexity of the model prevents analytical solutions), we use moment estimators based on the fitted smFISH dataset (Figure S18C). We find that while frequency changes are predicted accurately, the burst size is predicted accurately only for large means (Figure 4D). We find that specifically in the case of positive intercept (e.g., in the case of *IL1β*) the simple relationship does not reproduce the non-constant behavior at low mRNA levels. For the analyses of scRNA-seq datasets (Figures 4F and S19) we therefore fitted individual relationships separately (i.e. mean-variance, mean- burst-size, mean-frequency, etc.), rather than compare data with relationships predicted by the mean-variance line [Equation 8]. However, we then demonstrate that characteristics predicted by the theory are present in the fitted data, specifically there is a reciprocal relationship between burst size and frequency across considered genes (Figure S19F).

Intuitively, mean-variance relationships are expected to have zero intercepts. However, in both smFISH and scRNA-seq datasets we find evidence for both negative and positive intercepts. In the case of *TNFα* (negative intercept, Figure 4C), theoretical predictions of burst size and frequency based on the regression fit are consistent with fitted data and indeed predict a minimum in frequency changes. We find that in RAW 264.7 cells, there is always a basal (and substantial) expression of *TNFα* mRNA in unstimulated cells, which perhaps contributes to this behaviour (i.e. no true zero in the system). In general, fitted intercepts have relatively small values (comparing to the overall variance) and tend to be positive. This suggests elevated level of variance consistent with measurement noise (especially for small means). We accept that only a limited amount of data is available to be fitted per condition, thus individual fits may be affected by specific values of individual or groups of points. We consider these mean-variance relationships are empirical, and treat them as such in the manuscript.

#### Burstiness in Genomics Data

Inference of mean-variance relationship was performed using a dataset from BMDCs incorporating 29 scRNA-seq experiments (each corresponding to a single Fluidigm C1 experiment with up to 96 cells) on the response time-course (at 0, 1, 2, 3 and 6 h) as well as additional perturbations such as treatment with IFNβ, inhibition of paracrine secretion (chemical or physical on chip) or cell knockout for IFNR1 in and STAT1 expression (Shalek et al., 2014). We considered 812 genes that were induced by at least two-fold (compared to unstimulated cells) at the population level at any time point during the LPS stimulation [as identified in (Shalek et al., 2014)]. Visual inspection of the data revealed outliers in the linear regression fit, therefore, outlier removal method with Mahalanobis distance was used (with *0.05* threshold for outlier detection) (Finch, 2012). After removing low abundant genes (maximum mean expression <100 read counts) this resulted in 290 genes for the core TLR dataset (time-course) and 323 for the combined dataset (including perturbations). Bursting characteristics (based on moment estimators) for individual data points were fitted using linear regression and power functions (in semi log scale) when appropriate and presented as smooth curves. Robust regression (excluding data points with corresponding fitted residuals > 1.5⋅σ_residuals_) was used to either remove noisy data (as expected in the scRNA-seq measurement) or individual datapoints that did not affect the overall trend. Equations, fitted parameters, corresponding correlation coefficients and highlighted outliers are included in the Tables S3, S4, and S5. Fitting protocols were implemented in Python using R kernel, individual gene graphs were produced in MATLAB R2014a.

In order to validate estimates from scRNA-seq data, raw BAM dataset from Shalek et al. corresponding to LPS stimulation at 4h was downloaded and re-mapped using Picard Tools to remove duplicate reads (http://broadinstitute.github.io/picard/). Mapped data was normalised to read counts per million and compared with the original dataset (Figure S20). Specifically, for the set of LPS-dependent genes characterised by linear mean-variance relationships, mean and variance, as well as relative burst size and frequency (based on moment estimators) were calculated. In addition, chi-squared goodness-of-fit tests was performed to determine whether count data (in each dataset) follow negative binomial distribution. p-values were adjusted using Benjamini-Hochberg procedure for false discovery rate, genes with <10 non-zero reads (out of 95 captured cells) were not considered.

#### Statistical Analyses

Data are described by the sample mean and standard deviation (SD). Sample size are provided ion figure legends. All statistical analyses were performed in GraphPad Prism 8 or MATLAB. Data were checked for normality with the D’Agostino-Pearson omnibus test. When normal, parametric tests were performed (t-test, standard one-way ANOVA); otherwise, non-parametric tests are used (Mann-Whitney, Kruskal-Wallis ANOVA). Tukey’s or Dunn’s correction for multiple comparisons was applied, respectively. Contingency tables were assed with Fisher exact tests. MATLAB’s *chi2gof* chi-squared goodness-of-fit test was performed between count distributions and respective negative binomial distributions with parameters estimated from the data (using *fitdist* function). Benjamini and Hochberg method was used for multiple comparison adjustment in genomics data. Significance was defined for p-value (and adjusted p value, when relevant) <0.05. Details of all statistical tests are provided in the corresponding figure legends.

## Supplemental Information

Document S1. Figures S1–S23

Table S1. scRNA-Seq Analysis of Inducible TLR Gene Expression in RAW 264.7 Cells Stimulated with 500 ng/mL of Lipid A for 3 h, Related to Figure 1 Shown are normalized read counts for different gene subsets as well as the result of the correlation and clustering analyses.

Table S2. smFISH Probe Sets and Quantified mRNA Counts across All Experimental Conditions, Related to Figure 1

Table S3. Inference of the Mean-Variance Relationships for the LPS-Regulated Genes in BMDM Cells From (Shalek et al., 2014), Related to Figure 3 Shown are fitted linear regressions for the core TLR (time course measurement of responses to PAM, PIC, and LPS) and combined (with perturbations) datasets. Included are individual gene fits and data points, together with corresponding correlation coefficients (*R*^*2*^).

Table S4. Inference of the Transcriptional Bursting Characteristics for the LPS-Regulated Genes in BMDM Cells From (Shalek et al., 2014), Related to Figure 4 Shown are fitted relationships for (1) burst size versus mean; frequency versus mean; and noise versus mean using robust regression for the combined dataset; (2) comparison between fitted relationships for core and combined dataset using standard regression analyses. Bursting characteristics (based on moment estimators) were fitted using linear regression and power functions (in semi-log scale) when appropriate and presented as smooth curves. Included are model equations, individual data points, and gene fits with corresponding correlation coefficients (*R*^*2*^).

Table S5. Inference of the Transcriptional Bursting Characteristics for the LPS-Regulated Genes in BMDM Cells From (Shalek et al., 2014), Related to Figure 4 Shown are fitted relationship for variance versus burst size and variance versus frequency using robust regression for the combined dataset. Bursting characteristics (based on moment estimators) were fitted using linear regression and power functions (in semi log scale) when appropriate and presented as smooth curves. Included are model equations, individual data points, and gene fits with corresponding correlation coefficients (*R*^*2*^).

Document S2. Article plus Supplemental Information
