# Supplementary material for: Gene-Specific Linear Trends Constrain Transcriptional Variability of the Toll-like Receptor Signaling
Source: Cell Syst. 2020 Sep 23;11(3):300–314.e8. doi: 10.1016/j.cels.2020.08.007 (PMC7521480; doi:10.1016/j.cels.2020.08.007)
Supplement: Document S1. Figures S1–S23 [file mmc1.pdf]

Cell Systems, Volume *11*

Supplemental Information

Gene-Specific Linear Trends

Constrain Transcriptional Variability

# of the Toll-like Receptor Signaling

James Bagnall, William Rowe, Nissrin Alachkar, James Roberts, Hazel England, Christopher Clark, Mark Platt, Dean A. Jackson, Mark Muldoon, and Pawel Paszek

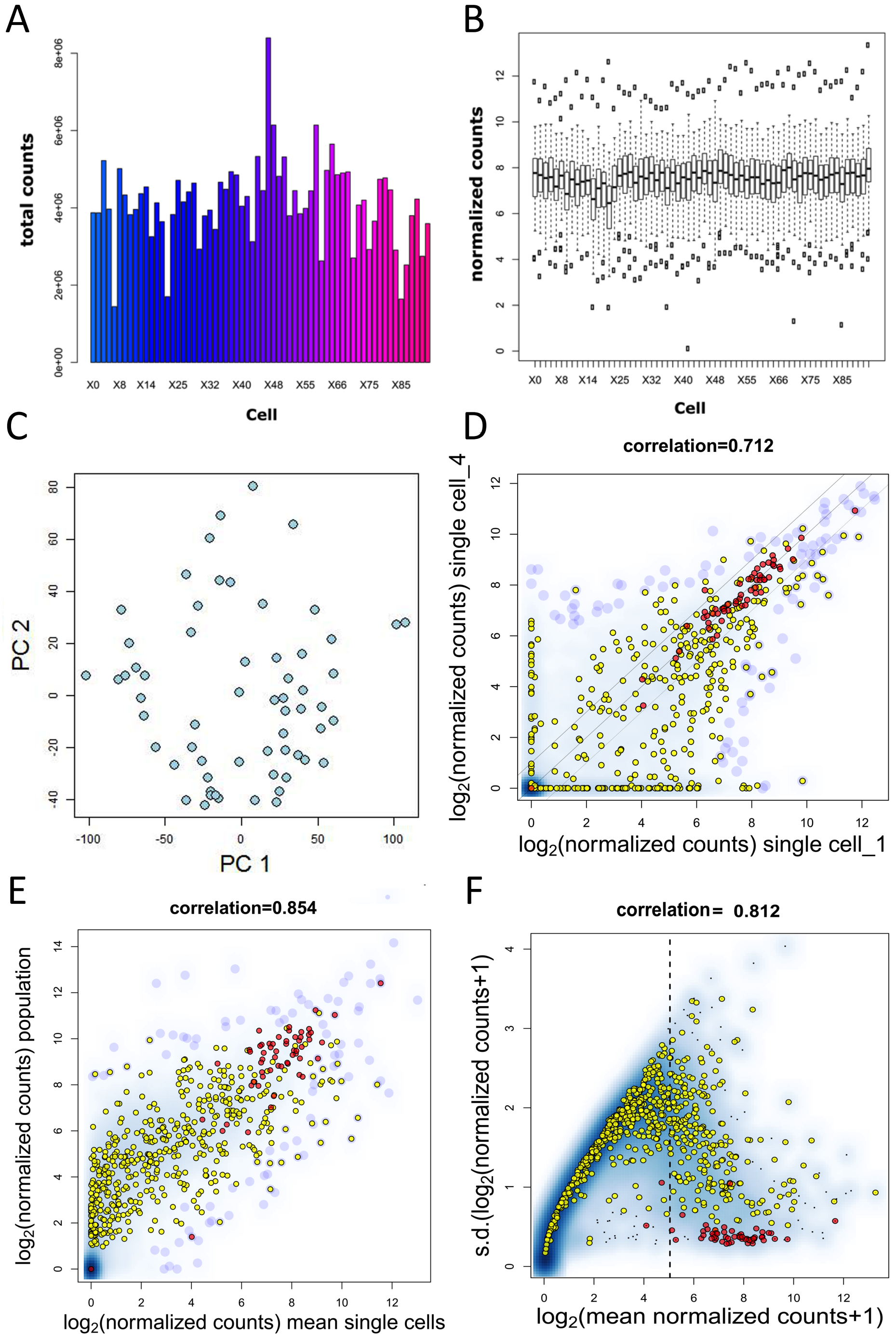

Figure S1. Variability within the scRNA-seq data, related to Figure 1. A. Total read counts across 64 cells before normalisation. 61 cells with read counts >*2x10*^{6} selected for subsequent analysis. **B**. Box-plots displaying the distribution of expression levels (in $\log_2$ scale) of housekeeping genes across single cells post normalisation by median count per cell (*x10*). **C**. PCA plot displaying variability between single-cell expression levels post normalisation. **D.** Normalized gene expression counts between two representative single cells. Shown is the smooth scatter plot, including lipid A response (in yellow), housekeeping genes (in red) and

other high confidence (HC) genes (in blue). Spearman rank coefficient indicates correlation between all genes between two cells. **E**. Comparison between transcript levels from population-level (from [1]) and mean of single cells. Shown is the smooth scatter plot, including lipid A response (in yellow), housekeeping genes (in red) and other genes (in blue). Spearman rank coefficient indicates correlation between all genes in the population (from [1]) and mean of single cells. **F.** Smooth scatter plot of standard deviation (SD) vs. mean normalized data. High confidence (HC) gene set defined for the expression level above cut-off line of $log_2(counts+1)=5$. Spearman rank coefficient indicates correlation between SD and mean normalized data (for all genes). Genes colour-coded as in D.

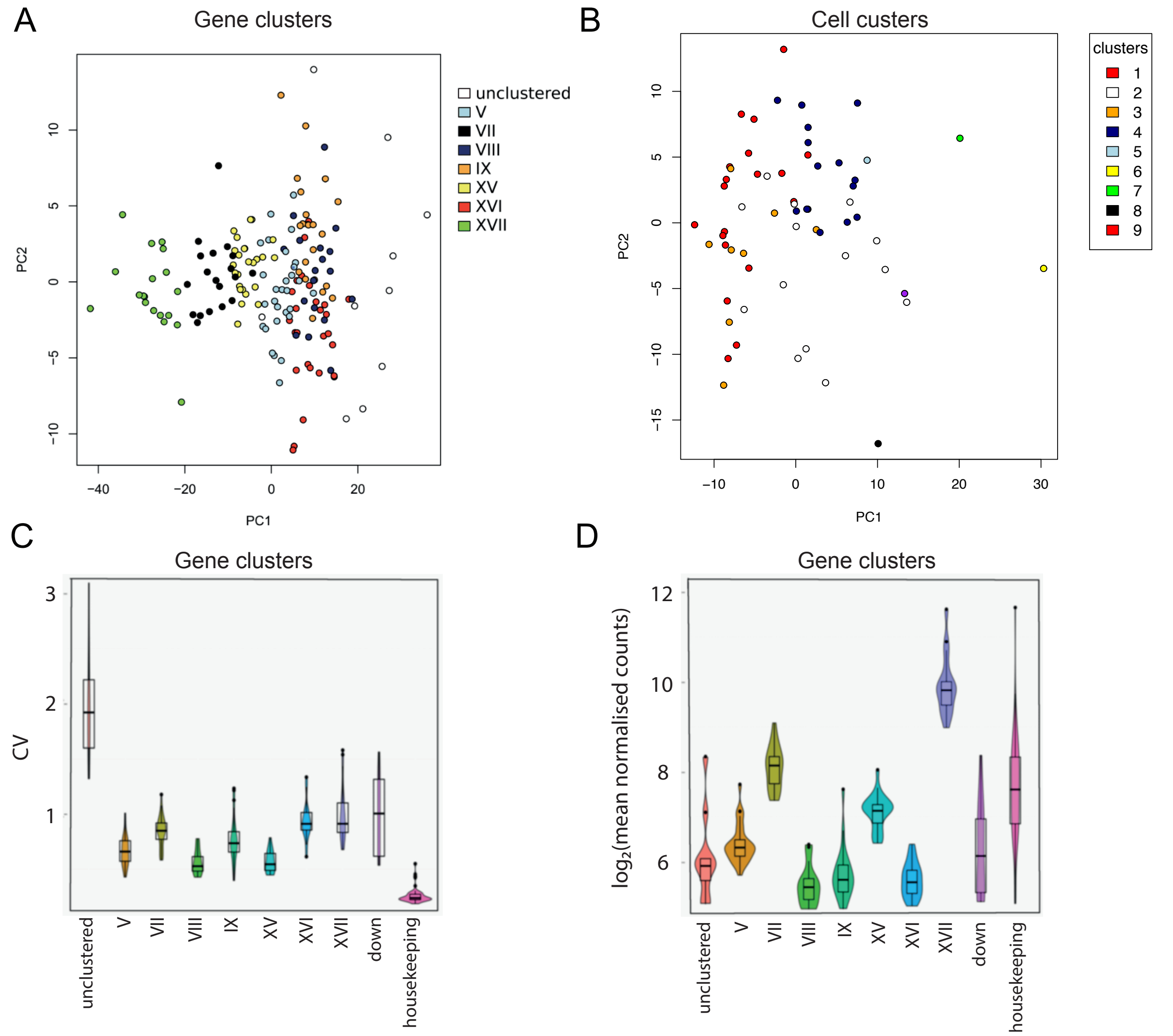

Figure S2. Clustering analysis of scRNA-seq data on lipid A-stimulated RAW 264.7 macrophages, related to Figure 1. A. Principal component analysis (PCA) of gene clusters from Fig. 1B. Shown are the first two principal components (PC1 vs. PC2). **B**. Principal component analysis of cell clusters identified in Fig. 1B. **C.** Violin plots displaying coefficient of variation (CV) of normalised transcript levels across gene clusters. **D.** Violin plots displaying mean normalized transcript levels across gene clusters.

A

group	TATA-box	total	p.value
un-clustered	8	10	<0.0001
V	3	28	1
VII	7	19	0.0045
VIII	2	17	1
IX	3	9	0.0793
XV	7	35	0.1784
XVI	6	27	0.1228
XVII	6	18	0.0143
Down-regulated	2	12	0.6424
housekeeping	15	55	0.0023
High-confidence set	227	1942	

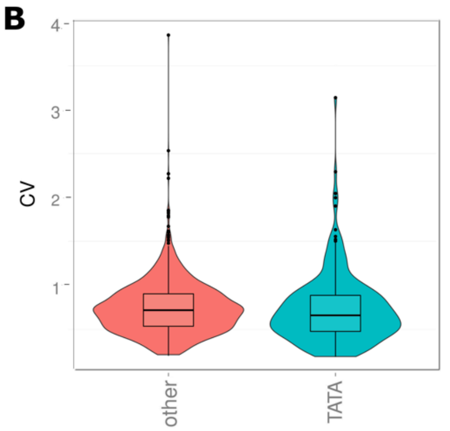

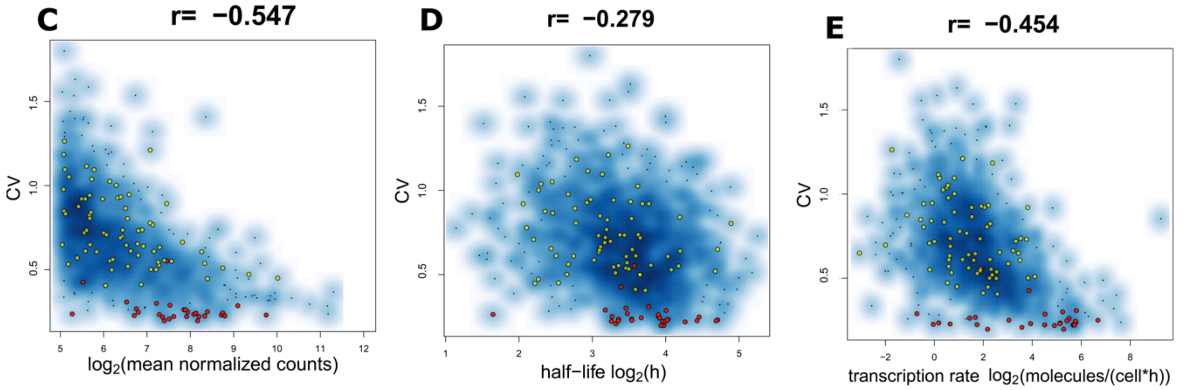

Figure S3. Physical properties of genes contribute to variation in gene expression measured in scRNA-seq data, related to Figure 1. A. Enrichment of TATA boxes the upstream regions of genes from clusters in Fig. 1B. **B.** Boxplot displaying coefficient of variation of genes with and without (proximal to) TATA-boxes (within the HC group). **C-E**. Coefficient of variation of individual genes against mean normalised transcript levels (C), mRNA half-life (D) and rate of transcription (E) (values taken from [2]). Shown are smooth scatter plots, including lipid A response (in yellow), housekeeping genes (in red) and other high confidence (HC) genes (in blue). Correlation coefficients calculated for all genes, assessed as statistically significant (p-val <0.001) according to Spearman rank correlation test.

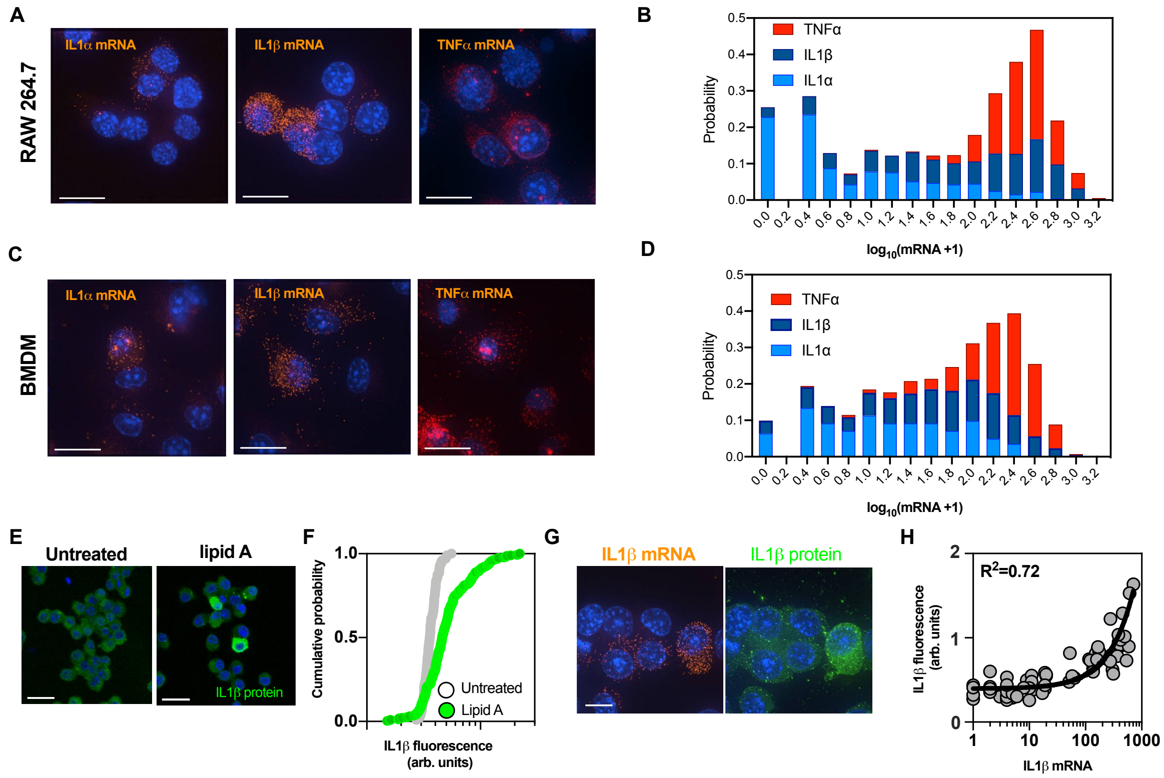

Figure S4. Analysis of TNFα, IL1α and IL1β expression, related to Figure 1. A. smFISH analysis of *IL1α*, *IL1β* and *TNFα* mRNA expression in RAW 264.7 cells. Shown are maximum intensity projections from deconvolved wide-field microscopy image z-stacks of representative cells stimulated with 500ng/ml lipid A for 3 h. mRNA transcript shown in orange, DAPI nuclear staining depicted in blue. Scale bar 10 µm. **B.** Distribution of mRNA counts from A. Shown are histograms for the *IL1α*, *IL1β* and *TNFα* abundances expressed as $\log_{10}$(mRNA+1) across at least three replicates, from 447, 718 and 356 cells, respectively. **C.** smFISH analysis of *IL1α*, *IL1β* and *TNFα* mRNA expression in BMDM cells. Shown are maximum intensity projections from deconvolved wide-field microscopy image z-stacks of representative cells stimulated with 500ng/ml lipid A for 3 h. mRNA transcript shown in orange, DAPI nuclear staining depicted in blue. Scale bar 10 µm. **D.** Histogram of mRNA counts in BMDMs (from C). Shown is analysis of 142, 732 and 322 cells for *IL1α*, *IL1β* ad *TNFα* across three replicates, respectively. **E**. Immunostaining of *IL1β* protein expression in RAW 264.7 macrophages. Shown are confocal microscopy images of cells treated with 500 ng/ml of lipid A for 3 h (or untreated). *IL1β* protein shown in green, DAPI nuclear staining depicted in blue. Scale bar 20 µm. **F**. Cumulative probability distribution of *IL1β* protein from A. Shown is analysis of 104 and 316 of untreated and lipid A-treated cells across three replicates, respectively. **G**. Dual smFISH and immunostaining analysis of *IL1β* mRNA and protein levels. Shown are deconvolved wide-field

microscopy images of representative RAW 264.7 cells stimulated with 500 ng/ml of lipid A for 3 h. mRNA transcript shown in orange (left panel), protein in green (right panel), DAPI nuclear staining depicted in blue. Scale bar 10 μm. **H**. Correlation between *IL1β* mRNA and protein levels in cells from C. Shown are individual cell counts depicted with circles, in black a nonlinear regression fit (with a coefficient of determination R^2).

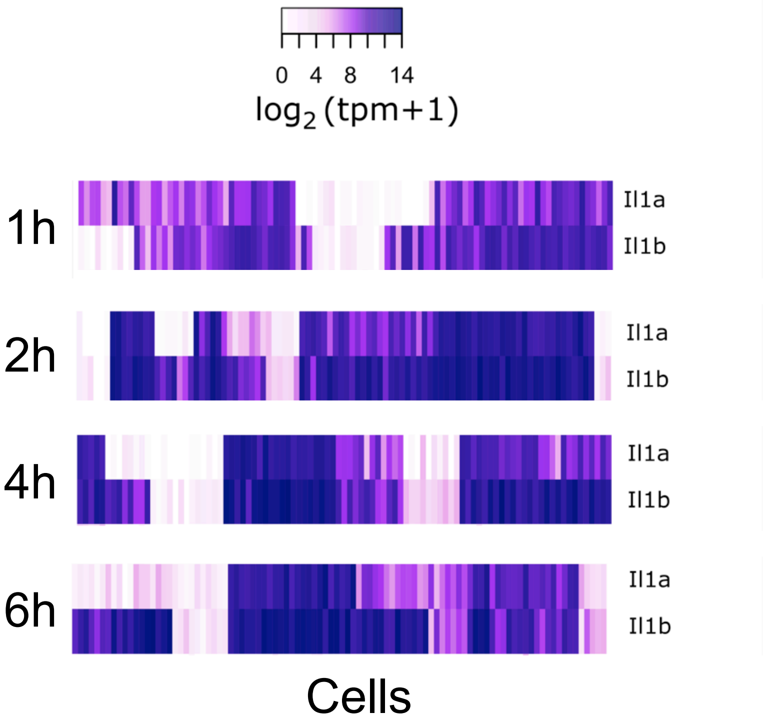

Figure S5. Analysis of *IL1α* and *IL1β* expression in mouse bone marrow derived dendritic cells, related to Figure 1. Heat maps displaying single cell expression of *IL1α* and *IL1β* across cells. Transcript levels measured as transcripts per million (tpm) were downloaded from the supplementary data from (Shalek et al., 2014). Expression levels are shown at 1, 2, 4 and 6 hours after LPS stimulation.

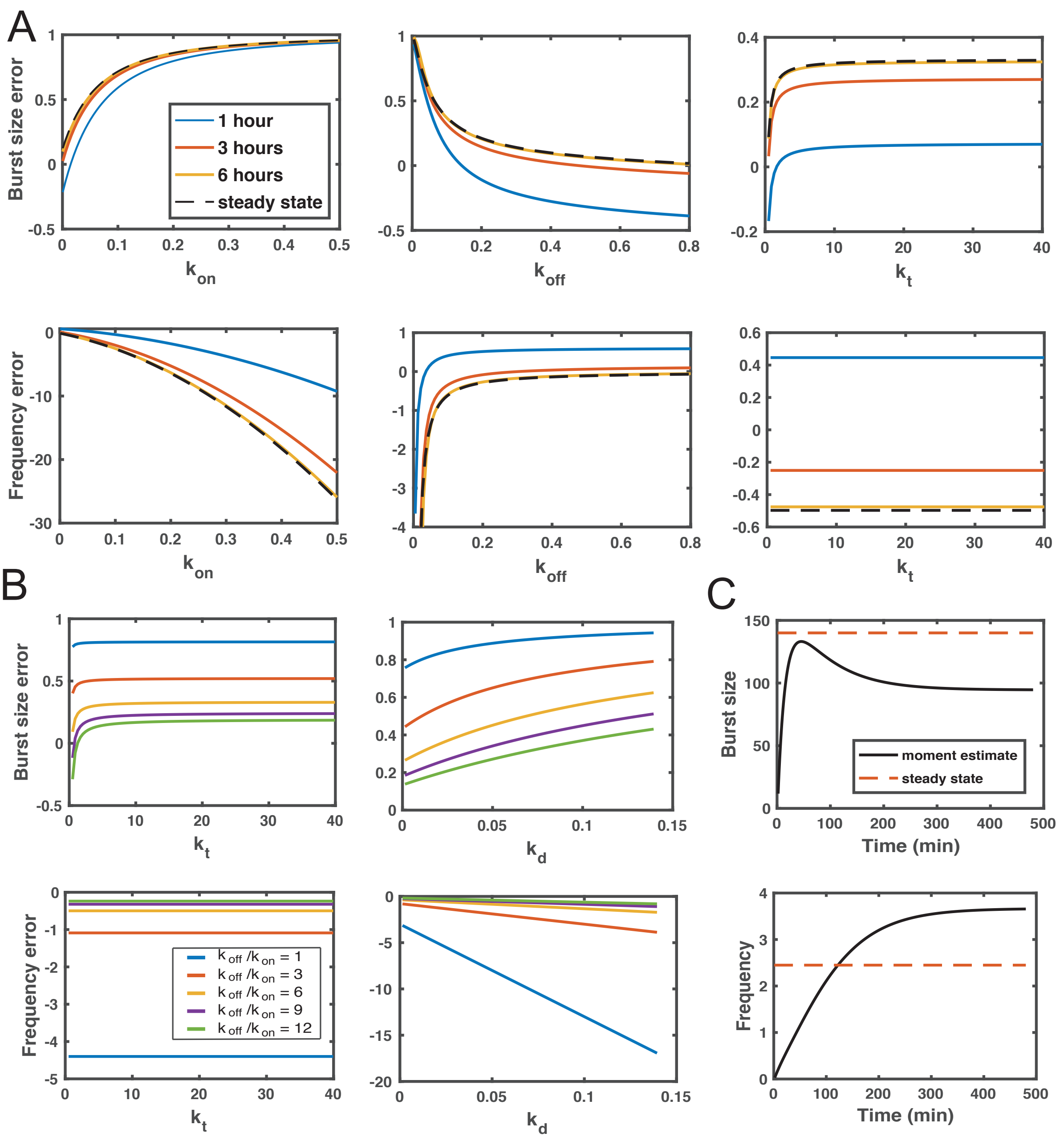

Figure S6. Comparison of point estimators of transcriptional bursting parameters, related to Figure 2. A. Fractional errors of moment estimators as a function of kinetic parameters. Shown are simulations using parameters of the fitted *TNFα* telegraph model (k_{on}=0.02 min^{-1}, k_{off}=0.12 min^{-1}, k_t=16.8 mRNA/min, k_d=0.014 min^{-1}) for systematic changes of individual parameter values (k_{on}, k_{off} and k_t, while retaining values of other). In colour lines, theoretical moment estimators using exact temporal mRNA distributions at 1, 3, 6 h; in broken lines steady-state errors given by Eqs (3) and (4). **B.** Steady-state burst size and frequency errors as a function of the 'burstiness'. Errors calculated for the parameters of the fitted TNFα model (k_{on}=0.02 min^{-1}, k_{off}=0.12 min^{-1}, k_t=16.8 mRNA/min, k_d=0.014 min^{-1}) for systematic changes of transcription (k_t) and degradation (k_d) rates. 'burstiness' defined as k_{off}/k_{on} ratio and

simulated by changing k_{off} rate (as highlighted), while maintaining k_{on} constant. **C.** Estimates of burst size and frequency for the fitted *TNF*α model at different time points (k_{on}=0.02 min^{-1}, k_{off}=0.12 min^{-1}, k_t=16.8 mRNA/min, k_d=0.014 min^{-1}). In broken lines are steady-state estimates using fitted kinetic parameters.

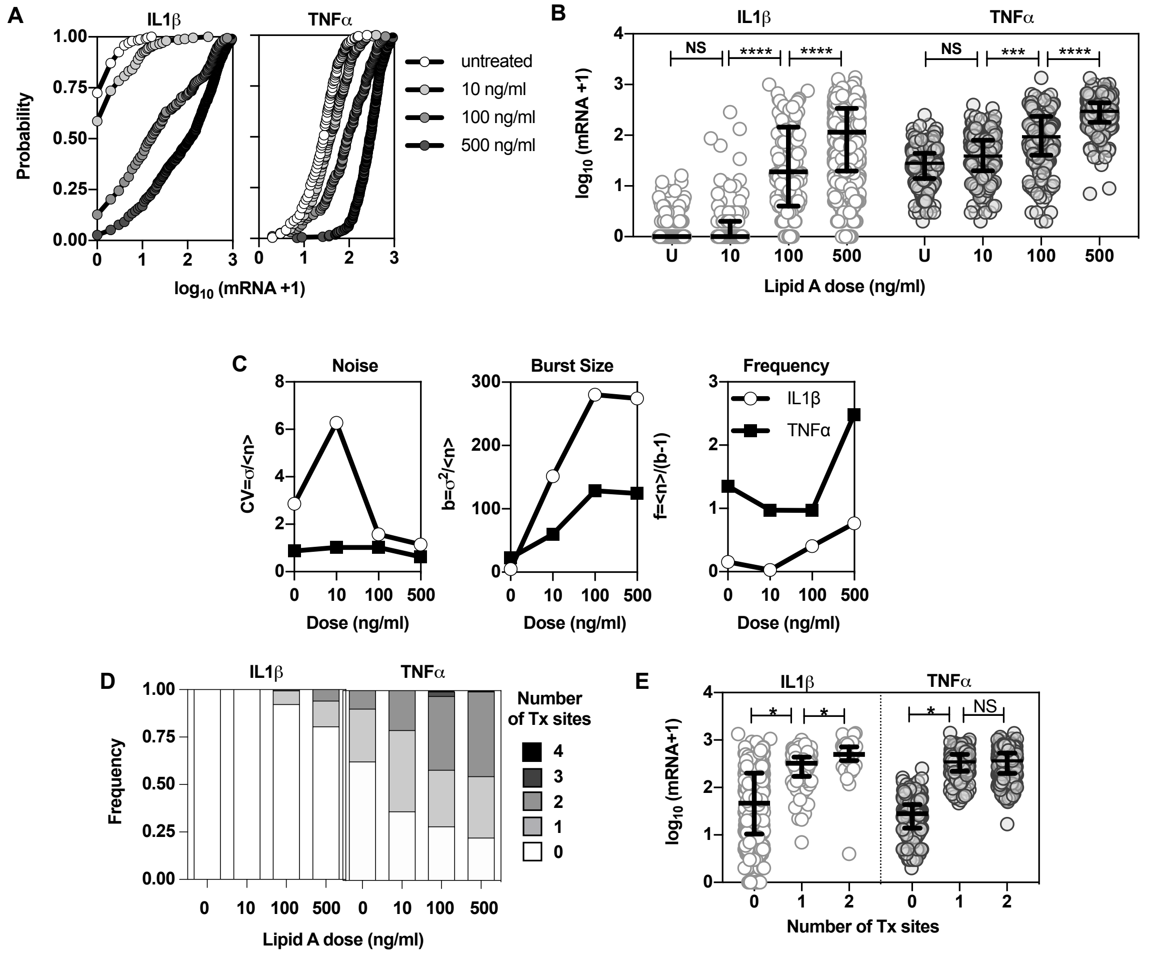

Figure S7. Dose-dependent regulation of *IL1β* and *TNFα* transcription in RAW 264.7 cells, related to Figure 2. A. Cumulative distribution function of mRNA counts in RAW 264.7 macrophages either untreated (U) or stimulated with 10, 100 and 500 ng/ml of lipid A for 3 h. 240, 208, 188 and 718 cells measured for *IL1β*, and 240, 208, 188 and 356 for *TNFα*, pooled across at least three smFISH experiments, respectively and expressed as $\log_{10}$(mRNA+1). **B**. Individual cell mRNA counts from A [with mean (and SD) per condition]. A nonparametric one-way ANOVA with Tukey's correction for multiple comparisons between groups summarised with ****- p-value <0.0001, ***- p-value <0.001, ** p-value <0.01, * p-value <0.05, NS- not significant. **C**. Coefficient of variation (*CV*), as well as moment estimators of burst size (*b*) and frequency (*f*) for mRNA count distributions from A. **D.** Distribution of transcription sites (Tx) in data from A. Shown is the fraction of cells with 0-4 transcription sites. **E.** mRNA abundance is correlated with the presence of transcription site. Shown are the *IL1β* and *TNFα* mRNA counts as a function of Tx number for cells stimulated with 500 ng/ml of lipid A (data from A). '*' denotes a statistical test (p-val<0.05) for a one-way ANOVA with Tukey's correction for multiple comparisons.

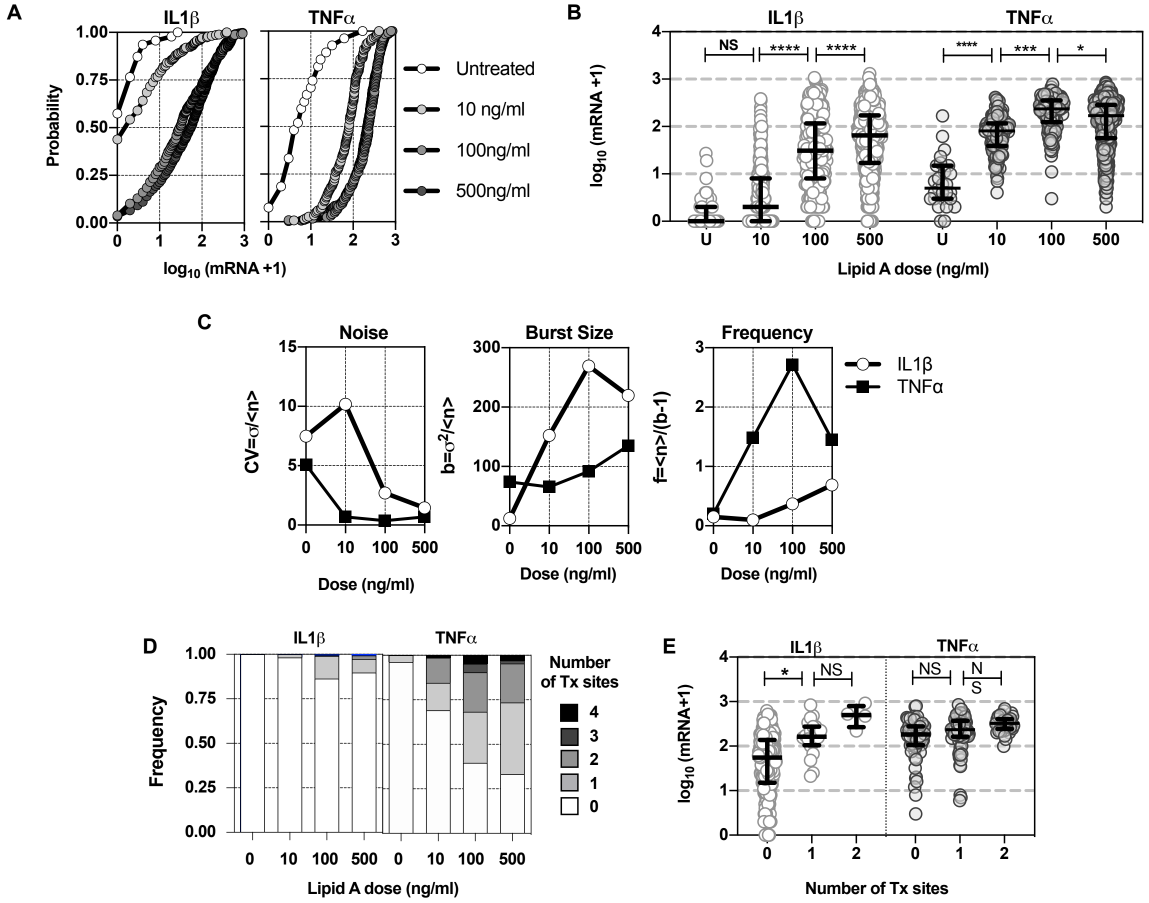

Figure S8. Dose-dependent regulation of *IL1β* and *TNFα* transcription in BMDMs, related to Figure 2. A. Cumulative distribution function of *IL1β* and *TNFα* mRNA counts in BMDMs either untreated (U, 0 ng/ml) or stimulated with 10, 100 and 500 ng/ml of lipid A for 3 h. 47, 276, 324 and 732 cells measured for *IL1β*, and 27, 149, 126 and 322 for *TNFα*, pooled across at least three smFISH experiments, and expressed as $\log_{10}$(mRNA+1). **B**. Individual cell mRNA counts from A. Shown are individual cell counts together with mean (and SD) per condition. Nonparametric Mann-Whitney U test for pairwise comparisons between groups summarised with ****- p-value <0.0001, ***- p-value <0.001, ** p-value <0.01, * p-value <0.05, NS- not significant. **C**. Characteristics of single cell mRNA expression. Shown is the coefficient of variation (*CV*) as well as moment estimators of burst size (*b*) and frequency (*f*) for mRNA count distributions from A. **D**. Distribution of transcription sites (Tx) in data from A. Shown is the fraction of cells with 0-4 transcription sites. **E**. *IL1β* and *TNFα* mRNA counts as a function of Tx number for cells stimulated with 500 ng/ml of lipid A (data from A). '*' denotes a statistical test (p-val<0.05) for one-way ANOVA with Tukey's correction for multiple comparisons.

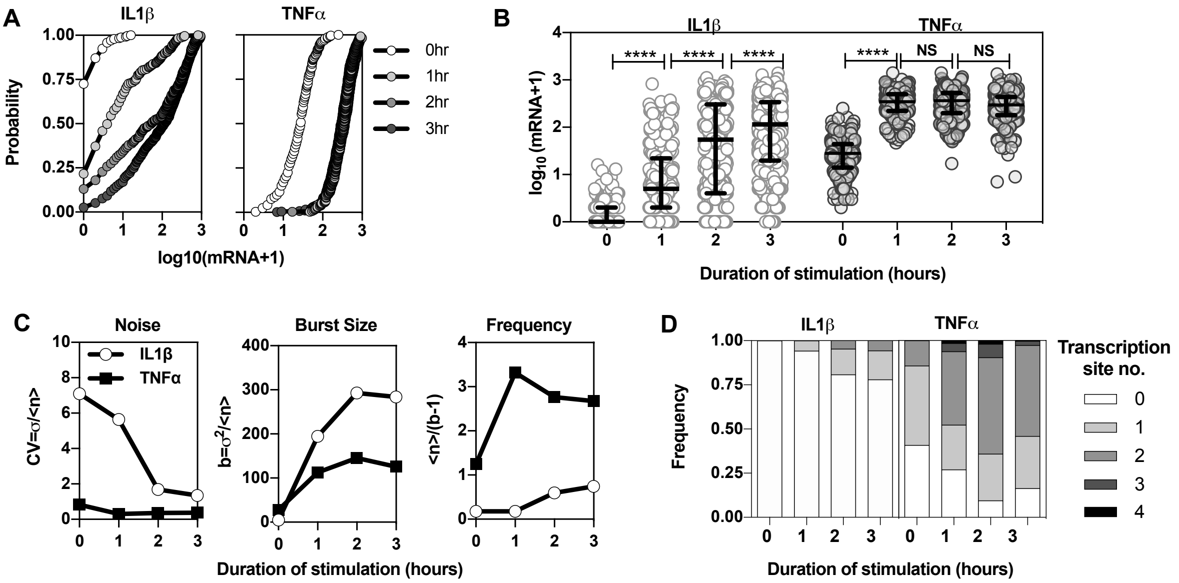

Figure S9. Variability of temporal *IL1β* and *TNFα* mRNA expression, related to Figure 2. A. Cumulative distribution function of mRNA counts in RAW 264.7 cells either untreated (0 h) or treated with 500 ng/ml lipid a for 1, 2 or 3 hours. Data pooled across at least three smFISH experiments and expressed as $\log_{10}$(mRNA+1). **B**. Individual cell mRNA counts in RAW 264.7 cells either untreated (0 h) or stimulated with 500 ng/ml of lipid A for 1, 2 and 3 h. 240, 253, 338 and 718 cells measured for IL1β, and 240, 253, 338 and 356 for TNFα, across at least three smFISH experiments, respectively. A nonparametric one-way ANOVA with Tukey's correction for multiple comparisons between groups summarised with ****- p-value <0.0001, ***- p-value <0.001, ** p-value <0.01, * p-value <0.05, NS- not significant. **C**. Characteristics of single cell mRNA expression. Shown is the coefficient of variation (*CV*) as well as moment estimators of burst size (*b*) and frequency (*f*) for mRNA count distributions from A. **D**. Distribution of transcription sites. Shown is the distribution of transcription sites in data from A. Shown is the fraction of cells with 0-4 transcription sites.

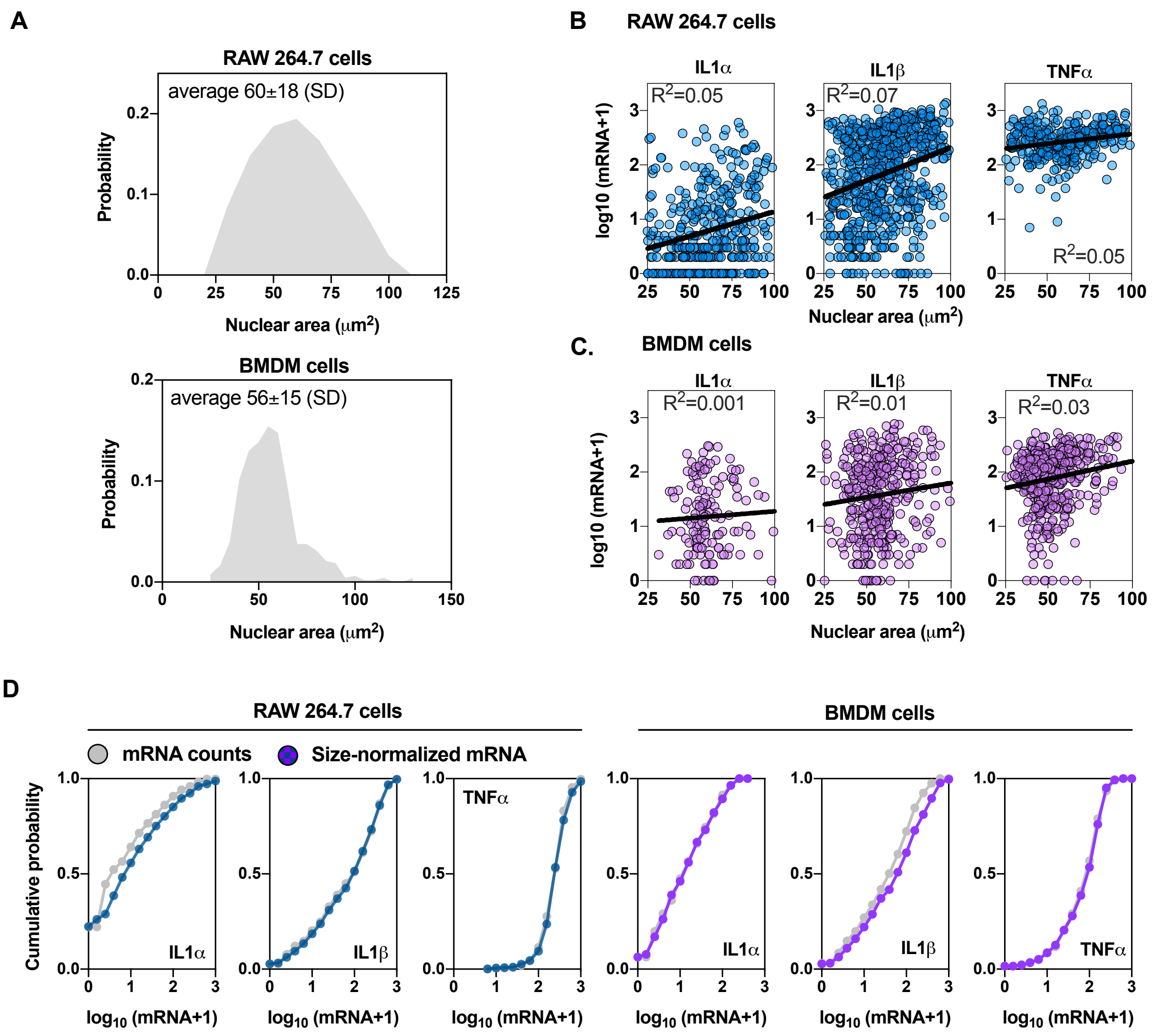

Figure S10. Effect of nuclear size on the smFISH count distribution, related to Figure 2. A. Smooth histogram of the nuclear area of RAW 264.7 and BMDM cells treated with 500 ng/ml lipid A for 3 h (data from Figs 1 and S8). Mean nuclear size and standard deviation (SD) displayed. **B and C.** Correlation between mRNA levels and the nuclear size. Shown are scatter plots across RAW 264.7 (B) and BMDM (C) cells stimulated with 500 ng/ml lipid A for 3 h (from data in Figs 1 and S8), with fitted regression line and fraction of variance explained by the nuclear size (correlation coefficient R^2). **D.** Cumulative probability distribution of the *IL1α*, *IL1β* and *TNFα* mRNA counts expressed as $\log_{10}$ (mRNA+1). Shown is the comparison between raw and cell size-normalized mRNA counts. Size-normalisation performed by scaling individual mRNA counts via the ratio of the average nuclear area in the population and nuclear size of particular cell.

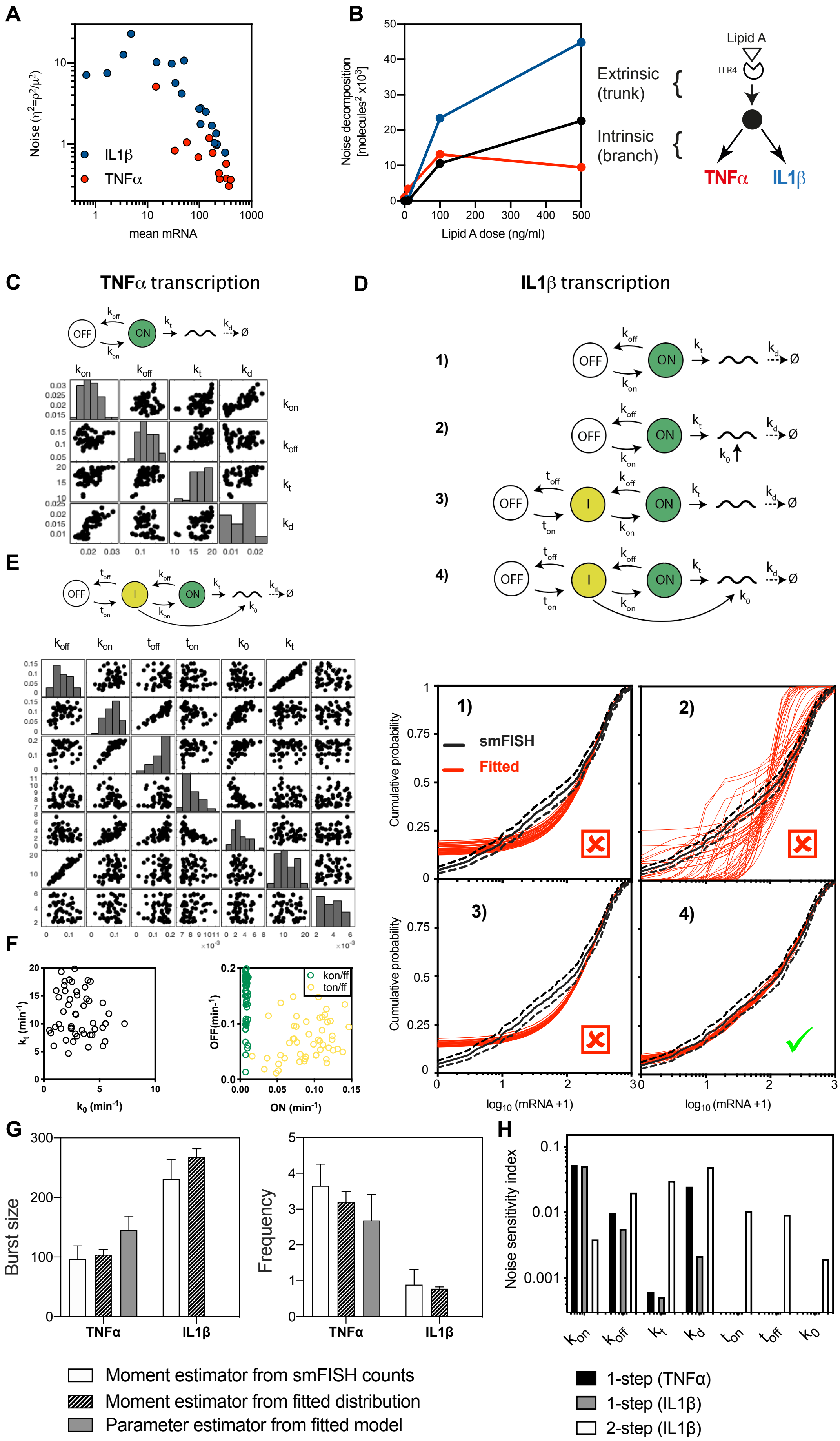

A
Noise (η²=σ²/μ²)
mean mRNA
IL1β
TNFα
B
Noise decomposition [molecules² x10³]
Lipid A dose (ng/ml)
Extrinsic (trunk)
Intrinsic (branch)
Lipid A
TLR4
TNFα
IL1β
C
TNFα transcription
D
IL1β transcription
E
F
G
H
smFISH
Fitted
Cumulative probability
log10 (mRNA +1)
Burst size
Frequency
Noise sensitivity index
Moment estimator from smFISH counts
Moment estimator from fitted distribution
Parameter estimator from fitted model
1-step (TNFα)
1-step (IL1β)
2-step (IL1β)

Figure S11. Mathematical model fits of *TNFα* and *IL1β*, related to Figure 2. A. Analysis of noise in the TNFα and IL1β smFISH counts. Show is η^2=CV^2 as a function mean mRNA expression (in $\log_{10}$ scale) combining all experimental conditions in this work (i.e., lipid A dose-response in RAW 264.7 and BMDM cells, time-course in RAW 264.7 and perturbations). **B**. Decomposition of noise in the *TNFα* and *IL1β* mRNA numbers; extrinsic noise (in black) versus intrinsic noise in *TNFα* (red) and *IL1β* (blue) levels calculated for the lipid A dose-response data (Fig. S8). Right: Schematic diagram of the noise decomposition. Trunk noise represents extrinsic variability between cells (potentially due to TLR signalling or generic transcriptional machinery), branch noise corresponds to gene specific intrinsic noise. **C**. Schematics of TNFα model shown alongside distribution of the fitted *TNFα* model parameters. Shown is a scatter plot matrix (with corresponding histograms) of individual 50 model fits from Fig. 2D (smFISH mRNA distribution at 3 h after 500 ng/ml lipid A stimulation in RAW 264.7 cells). **D**. *IL1β* transcription conforms to a two-step stochastic model. Top: Considered models of *IL1β* transcription: 1) one-step model with inducible transcription (k_t); 2) one-step model with a basal transcription (k_0); 3) basic two-step model; 4) two-step model. (Bottom) Shown is the comparison between measured and fitted *IL1β* mRNA distributions (3 h after 500 ng/ml lipid A treatment for the four different models in RAW 264.7 cells). In black: Kaplan-Meier estimator of measured CDF (with 95% confidence intervals), in red: a family of 50 models fitted to the data. **E**. Distribution of the fitted two-step model parameters from Fig. 2E (smFISH mRNA counts at 3 h after 500 ng/ml lipid A stimulation in RAW 264.7 cells). Shown is a scatter plot matrix (with histograms) of 50 individual model 4 fits (from B). **F**. *IL1β* model involves a combination of high and low transcription rates. Shown is the scatter plot of fitted transcription rates (k_t, k_0) for a family of 50 fitted models from Fig. 2E. (Right) Relationship between t_{on}/t_{off} rates (in yellow) and k_{on}/k_{off} rates (in green). **G**. Estimates of bursting characteristics for the fitted family of *TNFα* and *IL1β* models. Shown is the comparison between moment estimators for smFISH counts (from Fig. 2A), fitted distributions obtained with the model fits (from Fig. 2D and E) and estimates from based on fitted parameters values (for *TNFα).* **H.** Sensitivity analyses of different model structures**.** Shown is the local sensitivity analysis for the noise level (σ/μ) for the one-step *TNFα* and two-step *IL1β* models (as in Fig. 2D and E, respectively) as well as one-step model refitted to recapitulate mean and higher variance of *IL1β* expression (k_{on}=0.005 min^{-1}, k_{off}=0.05 min^{-1}, k_t=10 mRNA/min, k_d=0.003 min^{-1}). Sensitivity indexes calculated for 10% individual parameter changes.

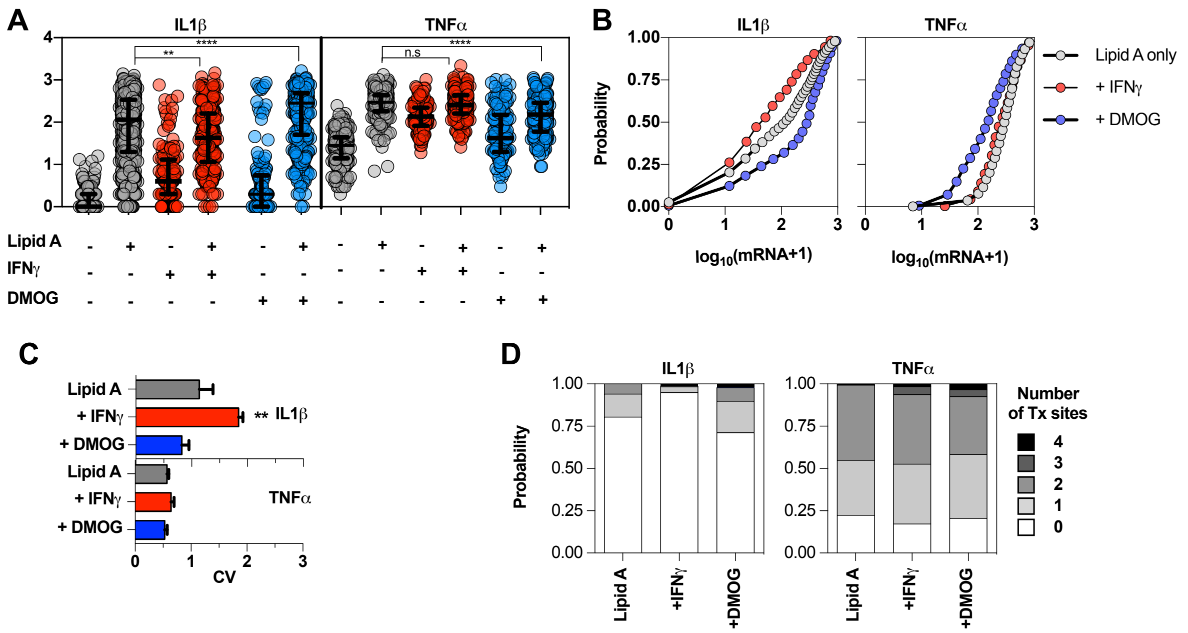

Figure S12. Perturbation of *TNFα* and *IL1β* gene expression by co-stimulation, related to Figure 3. A. smFISH analysis of *TNFα* and *IL1β* mRNA in response to co-stimulation with lipid A and DMOG or IFNγ. RAW 264.7 cells stimulated with 500 ng/ml of lipid A for 3 hours. For co-stimulation, cells were pre-treated with DMOG (0.5 mM) and IFNγ (100 ng/ml) for 24 hours before the treatment with lipid A. As a control, cells were stimulated with DMOG (0.5 mM) or IFNγ (100 ng/ml) for 27 hours. Nonparametric one-way ANOVA with Tukey's correction for multiple comparisons between lipid A-treated and co-stimulated groups summarised with ****- p-value <0.0001, **- p-value <0.01, ns- not significant. **B.** Cumulative distribution function of mRNA count data from A. **C.** Coefficient of variation (CV) for mRNA count data from A. '**' denotes significance <0.002 for a nonparametric one-way ANOVA with Tukey's correction for multiple comparisons. **D.** Distribution of transcription sites. Shown is the distribution of transcription sites in data A. Shown is the fraction of cells with 0-4 transcription sites.

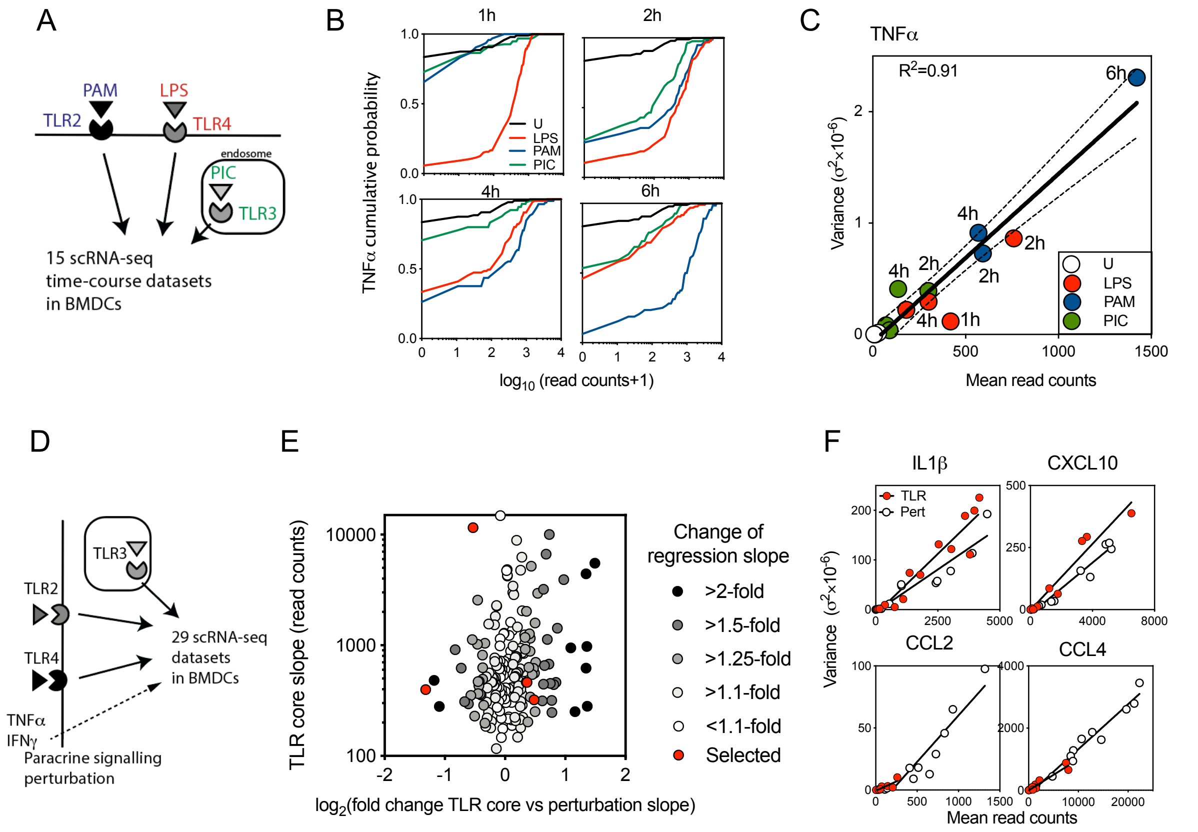

Figure S13. Analysis of TLR gene expression response heterogeneity in BMDCs, related to Figure 3. A. Schematic representation of the core TLR signalling network. **B.** Temporal TNFα expression patterns are treatment specific. Shown are cumulative distribution functions of TNFα read counts across different treatments [untreated (U), LPS, PAM and PIC, as indicated on the graph). Distributions estimated from the scRNA-seq data from (Shalek et al., 2014). **C**. The inferred linear regression trend (with 95% confidence intervals) for TNFα from [3] Differential TLR stimulation colour-coded as in A, highlighted are specific measurement times. Coefficient of determination depicted with R^2. **D.** Schematic representation of TLR paracrine signalling pathways. BMDC either stimulated with core TLR treatments, or perturbed using generic (e.g. Golgi inhibitors) and specific paracrine signalling modulators, e.g. using INFAR1, TNFR and STAT1 knockout cells. **E.** Effect of paracrine signalling perturbation on the fitted mean-variance regression trends. Shown is the fold-change of the regression slopes fitted for core TLR dataset vs. set of signalling perturbations (in $\log_2$) for 195 high confidence genes (defined by $R^2>0.75$ for both regression fits). Fold-change levels depicted in grey scale (as indicated on the graph). **F.** Fitted mean-variance relationships for selected genes from D. Data points corresponding to core TLR and perturbation indicated with red and open circles, respectively. Gene-specific regression slopes are statistically different (p-value <0.05) as assessed with a Student t-test.

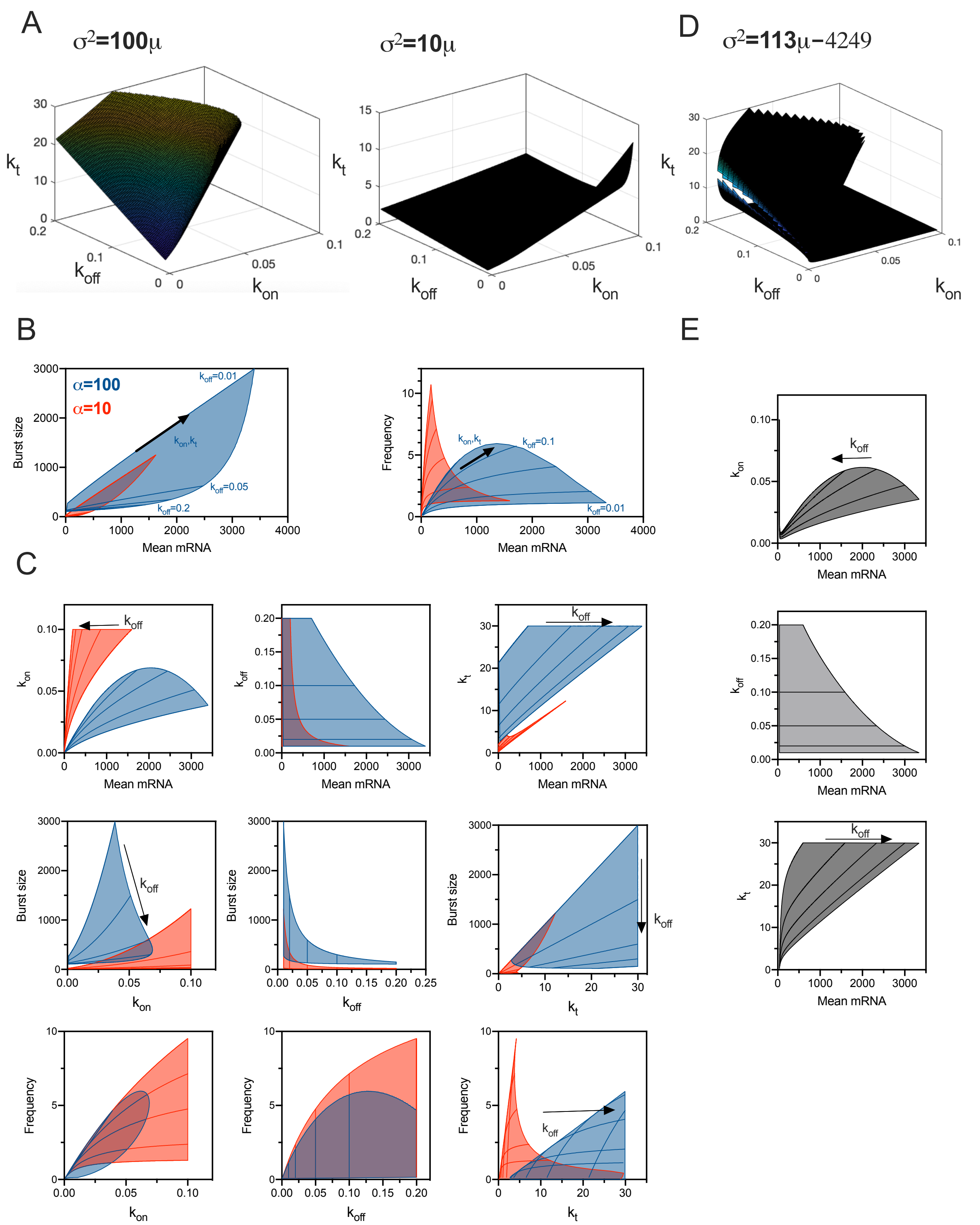

Figure S14. Linear mean-variance relationships constrain parameters of transcription, related to Fig. 4. A. Three-dimensional (k_{off}, k_{on}, k_t) parameter surface on which the $\sigma^2 = \alpha\mu$ relationship holds. Calculation performed using Eq. (6) for biologically plausible set of gene activity switching rates, $k_{off}<0.2\ min^{-1}$ and $k_{on}<0.1\ min^{-1}$, while assuming $k_d = 0.014\ min^{-1}$ (corresponding to fitted *TNFα* mRNA degradation rate) and $k_t<30\ min^{-1}$. Shown are relationships for $\alpha=100$ and $\alpha=10$, as highlighted on the graph. **B.** Burst size and burst

frequency as function of the mean mRNA response calculated for the kinetic parameter values given by surface from A. In different colours are the feasible parameter ranges (blue for $\alpha=100$, red for $\alpha=10$), thin lines correspond to $k_{off}=0.01, 0.02, 0.05, 0.1, 0.2\ min^{-1}$. **C**. Bursting characteristics as a function of kinetic parameter values given by surface from A. In different colours are the feasible parameter ranges (blue for $\alpha=100$, red for $\alpha=10$), thin lines correspond to $k_{off}=0.01, 0.02, 0.05, 0.1, 0.2\ min^{-1}$. **D.** Three-dimensional (k_{off}, k_{on}, k_t) parameter surface on which the $\sigma^2=\alpha\mu-\alpha_0$ relationship holds. Calculation performed using Eq. (7) for $\alpha=113$ and $\alpha_0=4249$ (corresponding to the fitted *TNF*α relationship) for biologically plausible set of gene activity switching rates, $k_{off}<0.2\ min^{-1}$ and $k_{on}<0.1\ min^{-1}$, while assuming $k_d=0.014\ min^{-1}$ and $k_t<30\ min^{-1}$. Highlighted lines correspond to $k_{off}=0.01, 0.02, 0.05, 0.1, 0.2\ min^{-1}$. **E**. Kinetic parameter values as a function of mean mRNA expression given by surface in D.

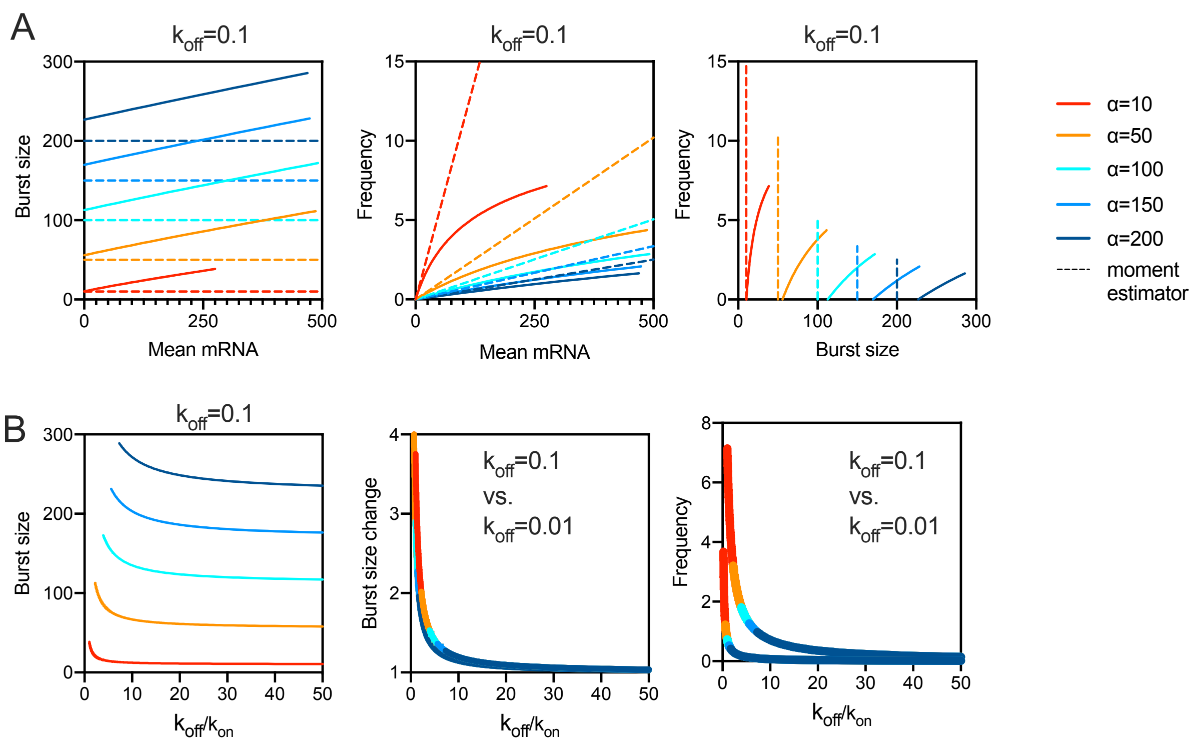

Figure S15. Reciprocal relation between the burst size and frequency, related to Fig. 4. A. Changes of burst size and frequency across the underlying range of mean mRNA expression. Calculation performed using Eq. (6) for biologically plausible set of gene activity switching rates $k_{on}<0.1\ min^{-1}$, while assuming $k_d=0.014\ min^{-1}$ (corresponding to fitted *TNFα* mRNA degradation rate), $k_t<30\ min^{-1}$ and μ<500. Fife putative genes are considered, each with a different regression slope (α, in different colour lines). Shown are relative frequency and burst size changes per α, corresponding to $k_{off}=0.1\ min^{-1}$. In broken lines are the moment estimators (i.e., '*bursty*' regime). B**.** Analysis of absolute burst size and frequency from B as a function of k_{off}/k_{on} ratio. Colour coding as in A. Calculations performed for $k_{off}=0.1$ *and* $0.01\ min^{-1}$.

A

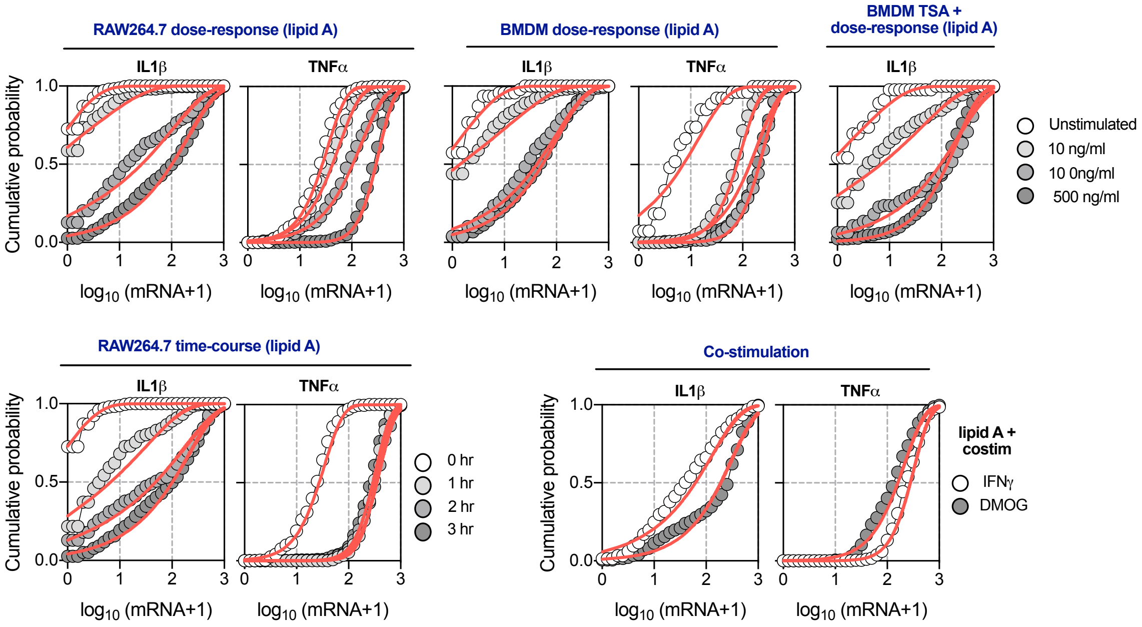

B

		TNFα			IL1β		
**Cell Line**	**Condition**	**r**	**p**	**NB p-val**	**r**	**p**	**NB p-val**
RAW264.7	Unstimulated	1.56	0.04	0.25	0.23	0.26	N/A
	Lipid A (3 hrs; 10 ng/ml)	1.18	0.02	0.09	0.14	0.03	N/A
	Lipid A (3 hrs; 100 ng/ml)	0.93	0.01	0.17	0.30	0.00	0.014
	Lipid A (3 hrs; 500 ng/ml)	2.60	0.01	0.29	0.54	0.00	<0.001
	Lipid A (1 hr; 500 ng/ml)	3.25	0.01	0.33	0.26	0.01	0.024
	Lipid A (2 hrs; 500 ng/ml)	2.53	0.01	0.02	0.32	0.00	<0.001
	IFNγ	1.81	0.01	0.12	0.24	0.01	N/A
	IFNγ + Lipid A	2.19	0.01	0.02	0.51	0.00	0.014
	DMOG	0.67	0.01	0.003	0.10	0.00	N/A
	DMOG + Lipid A	1.24	0.01	0.09	0.76	0.00	<0.001
BMDM	Unstimulated	0.52	0.03	N/A	0.25	0.13	N/A
	Lipid A (3 hrs; 10 ng/ml)	1.67	0.02	0.24	0.17	0.01	0.069
	Lipid A (3 hrs; 100 ng/ml)	2.04	0.01	0.03	0.46	0.00	0.003
	Lipid A (3 hrs; 500 ng/ml)	1.80	0.01	0.051	0.58	0.01	0.586
	TSA	N/A	N/A	N/A	0.20	0.05	N/A
	TSA + Lipid A (3 hrs; 10 ng/ml)	N/A	N/A	N/A	0.23	0.00	0.002
	TSA + Lipid A (3 hrs; 100 ng/ml)	N/A	N/A	N/A	0.48	0.00	0.027
	TSA + Lipid A (3 hrs; 500 ng/ml)	N/A	N/A	N/A	0.88	0.00	0.196

Figure S16. Negative binomial fits of the measured *IL1β* and *TNFα* mRNA distributions, related to Fig 4. A. Comparison between negative binomial fit (in red) and measured mRNA distributions (depicted with different colour dots) across all smFISH datasets. **B.** Fitted negative binomial parameters (r and p) across different conditions. P-values denote result for a chi-squared test for the smFISH count distribution following negative binomial with respective parameters. N/A denotes cases when the test cannot be performed (due to low count levels) or measurement is not obtained.

A

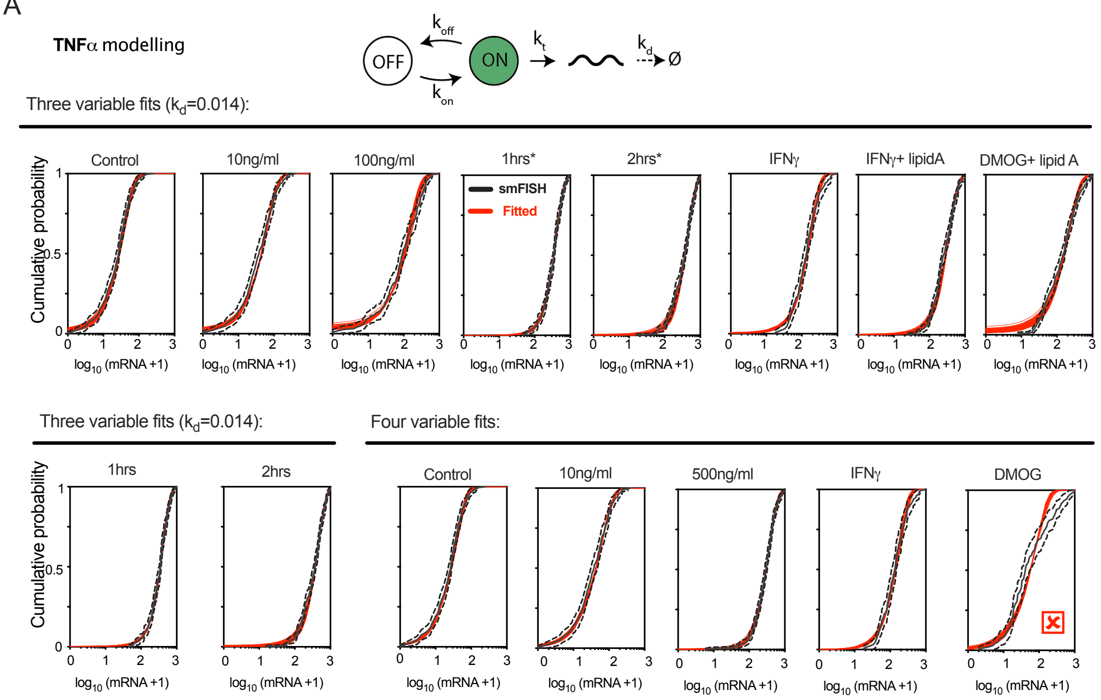

B

	kon	SD	koff	SD	kt	SD	kd	SD
Control	0.013	0.001	0.145	0.040	3.088	0.831	0.014	NA
10ng/ml	0.011	0.001	0.158	0.032	6.186	1.250	0.014	NA
100ng/ml	0.009	0.001	0.143	0.032	16.148	2.830	0.014	NA
500ng/ml	0.021	0.004	0.119	0.024	16.818	2.125	0.014	0.004
1 hrs*	0.023	0.002	0.110	0.030	16.400	2.747	0.014	NA
2 hrs*	0.020	0.003	0.082	0.024	15.908	2.514	0.014	NA
IFNy	0.016	0.001	0.163	0.028	14.065	2.537	0.014	NA
IFNy +lipid A	0.019	0.002	0.115	0.019	17.794	1.730	0.014	NA
DMOG + lipid A	0.011	0.001	0.112	0.025	16.998	2.391	0.014	NA
Control	0.028	0.007	0.156	0.029	4.510	0.785	0.042	0.012
10ng/ml	0.022	0.006	0.171	0.024	9.296	1.460	0.039	0.011
IFNy	0.027	0.006	0.154	0.027	16.696	2.220	0.032	0.009
DMOG	0.021	0.005	0.174	0.023	12.738	2.229	0.037	0.009
1 hrs	0.052	0.004	0.152	0.021	18.639	0.987	0.014	NA
2 hrs	0.024	0.003	0.095	0.020	17.398	1.914	0.014	NA

C

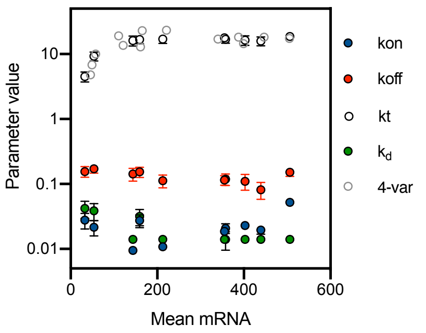

D

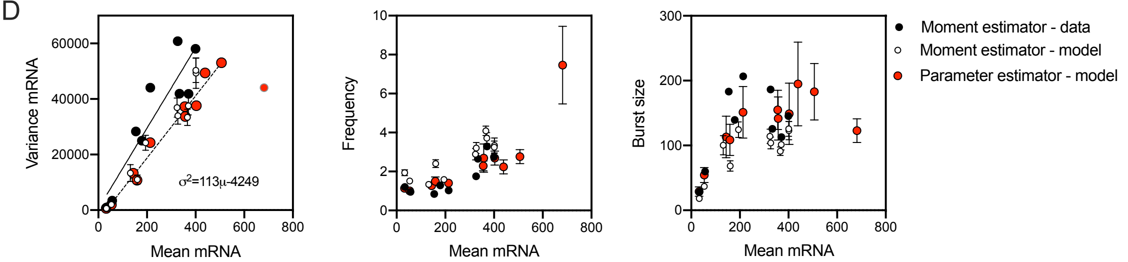

Figure S17. Model analysis of *TNFα* distributions, related to Figure 4. A. The comparison between measured and fitted *TNFα* mRNA distributions across all experimental conditions in RAW 264.7 cells (including dose- and time-response as well as perturbations). In black: Kaplan-Meier estimator of measured CDF (with 95% confidence intervals), in red: a family of 50 two-step models fitted to the data. Subset of conditions fitted assuming fixed k_d (three variable fits). * denotes data collected at 1 or 2 h, but fitted at 3 h after stimulation. **B**. Summary of fitted parameter values (mean and standard deviation, SD) from A. **C.** Parameter values as

a function of the fitted mean mRNA expression per condition (using theoretical steady-state levels). **D**. Estimates of variability and bursting characteristics for the fitted *TNF*α models. Shown is the comparison between moments and moments estimators for smFISH counts (in black) and mRNA distributions obtained with the model fits (with open circles). Also shown are estimators based on fitted kinetic parameter rates (in red). Characteristics represented as function of the corresponding mean values. Regression lines fitted to mean-variance data for smFISH counts (solid line) and steady-state mean/variance calculated for fitted model parameters (broken line, with the corresponding equation displayed).

A

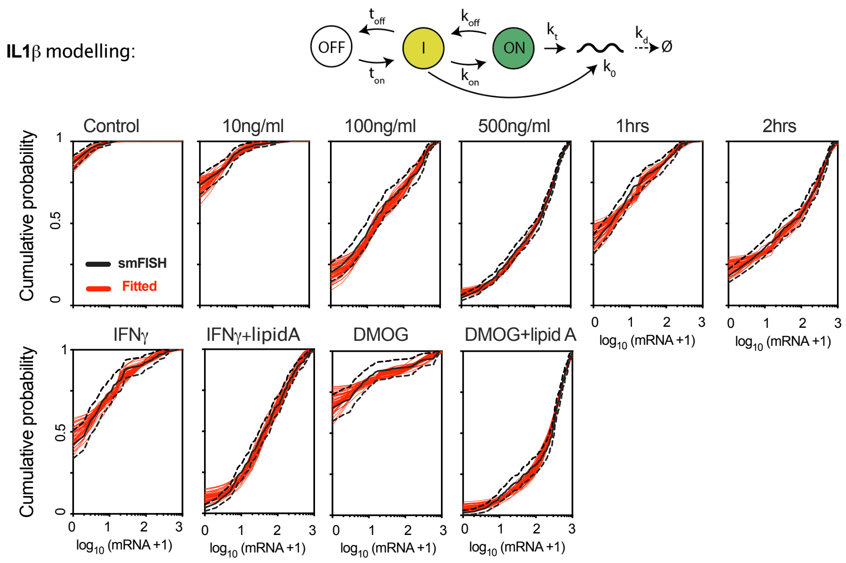

B

	ton	SD	toff	SD	kon	SD	koff	SD	k0	SD	kt	SD	kd	SD
Control	0.002	0.002	0.153	0.053	0.053	0.061	0.126	0.074	0.262	0.208	1.021	2.397	0.0037	NA
10ng/ml	0.002	0.003	0.146	0.052	0.029	0.027	0.124	0.060	0.664	0.492	5.833	5.731	0.0037	NA
100ng/ml	0.006	0.001	0.160	0.035	0.060	0.022	0.073	0.041	2.154	1.059	13.492	5.156	0.0037	NA
500ng/ml	0.008	0.001	0.144	0.045	0.091	0.031	0.072	0.036	3.054	1.513	11.683	4.108	0.0038	0.001
1hrs	0.010	0.003	0.154	0.038	0.062	0.024	0.114	0.048	1.202	0.595	11.864	4.210	0.0037	NA
2 hrs	0.007	0.002	0.136	0.038	0.130	0.038	0.081	0.050	3.492	2.308	13.148	4.734	0.0037	NA
IFNy	0.003	0.001	0.160	0.039	0.035	0.019	0.083	0.052	1.235	0.659	11.150	5.055	0.0037	NA
IFNy+ lipid A	0.008	0.001	0.147	0.045	0.044	0.019	0.075	0.038	2.710	1.359	13.966	4.460	0.0037	NA
DMOG	0.002	0.001	0.152	0.038	0.071	0.036	0.045	0.034	1.781	1.538	14.240	4.763	0.0037	NA
DMOG + lipid A	0.011	0.004	0.141	0.040	0.125	0.040	0.090	0.039	3.316	2.438	12.704	3.870	0.0037	NA

C

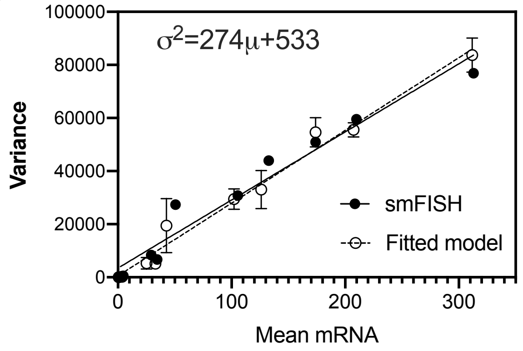

D

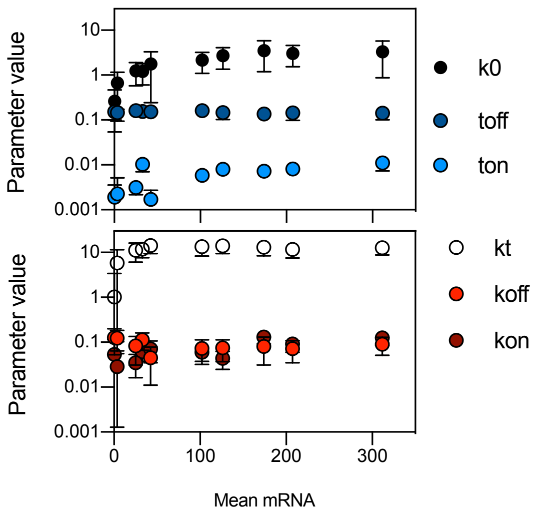

Figure S18. Model analysis of *IL1β* distributions, related to Figure 4. A. The comparison between measured and fitted *IL1β* mRNA distributions across all experimental conditions in RAW 264.7 cells (including does and time-response as well as perturbation). In black: Kaplan-Meier estimator of measured CDF (with 95% confidence intervals), in red: a family of 50 two-step models fitted to the data. **B**. Summary of fitted parameter values (mean and standard deviation, SD) from A. Models were fitted assuming fixed k_d (except of 500 ng/ml lipid A stimulation). **C**. Mean-variance relationship for the fitted family of *IL1β* models. Shown is the

comparison between moments for smFISH counts and mRNA distributions obtained with the model fits (with SDs). Regression lines fitted to smFISH counts (full circles) and mean/variance calculated for fitted model distributions (open circles with the corresponding equation displayed). **D.** Parameter values as a function of the fitted mean mRNA expression per condition.

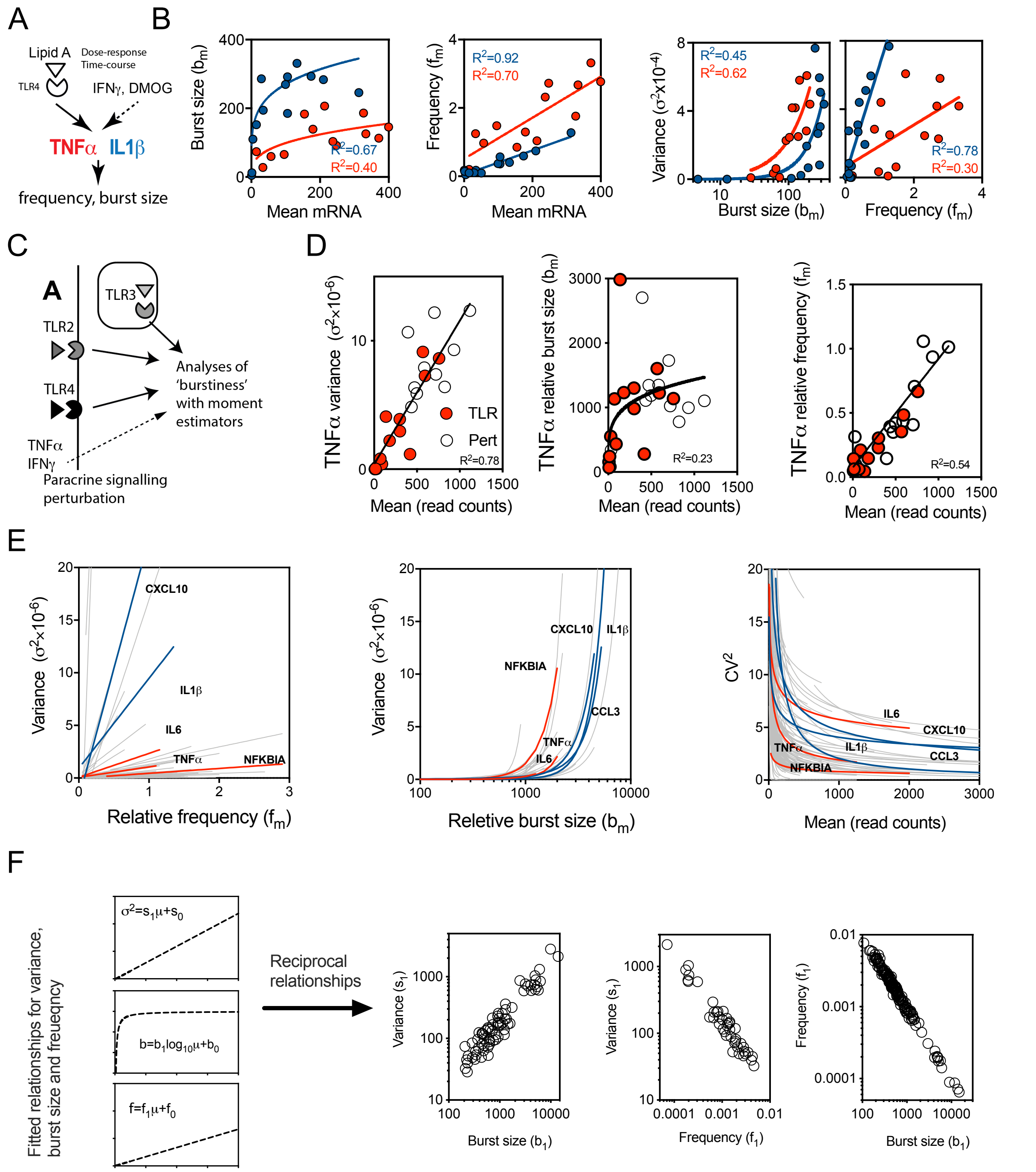

Figure S19. Analysis of transcriptional bursting using moment estimators, related to Figure 4. A. Schematic representation of immune-modulating pathways used to assay TNFα and IL1β mRNA expression. **B**. Moment estimators of transcriptional bursting in the smFISH dataset. Shown are individual data points and fitted relationships (in blue and red for *IL1β* and *TNFα*, respectively) for smFISH data (dose-response, time-course, as well as IFNγ and DMOG perturbation for BMDMs and RAW 264.7 macrophages). Power functions used to fit burst size (otherwise linear regression was applied). Coefficient of determination depicted with R^2 (colour coded for the respective gene). **C**. Schematic representation of the TLR and paracrine

signalling pathways. **D**. Transcriptional bursting of *TNF*α in the 26 scRNA-seq datasets in BMDCs from [3]. Shown is the fitted relationships combining core TLR (in red circles) and perturbation (in open circles) datasets using relative moment estimators (based on read counts). Coefficient of determination depicted with R^2. **E**. Fitted relationships for the variance and noise of TLR-induced genes, for the combined core TLR and perturbation dataset from [3]. Left: Relative frequency-variance relationship for the 112 genes (defined by Spearman correlation coefficient $R^2>0.5$) obtained using robust linear regression fit (with intercept). Middle: Relative burst size-variance relationship for the 189 genes (defined by Spearman correlation coefficient $R^2>0.68$) obtained using robust power series fit ($log_{10}(\sigma^2)=p1+p2\cdot \mu^{p3}$). Right: Mean-noise ($CV^2$) relationship for the 180 genes (defined by Spearman correlation coefficient $R^2>0.70$) obtained using robust curve fitting ($CV^2=p1\cdot \mu^{p2}$). **F.** Reciprocal relationship between variance, burst size and frequency in the fitted bursting characteristics (from Fig. 4F). Shown are pairwise scatter plots between fitted regression coefficients (as depicted on the left panel).

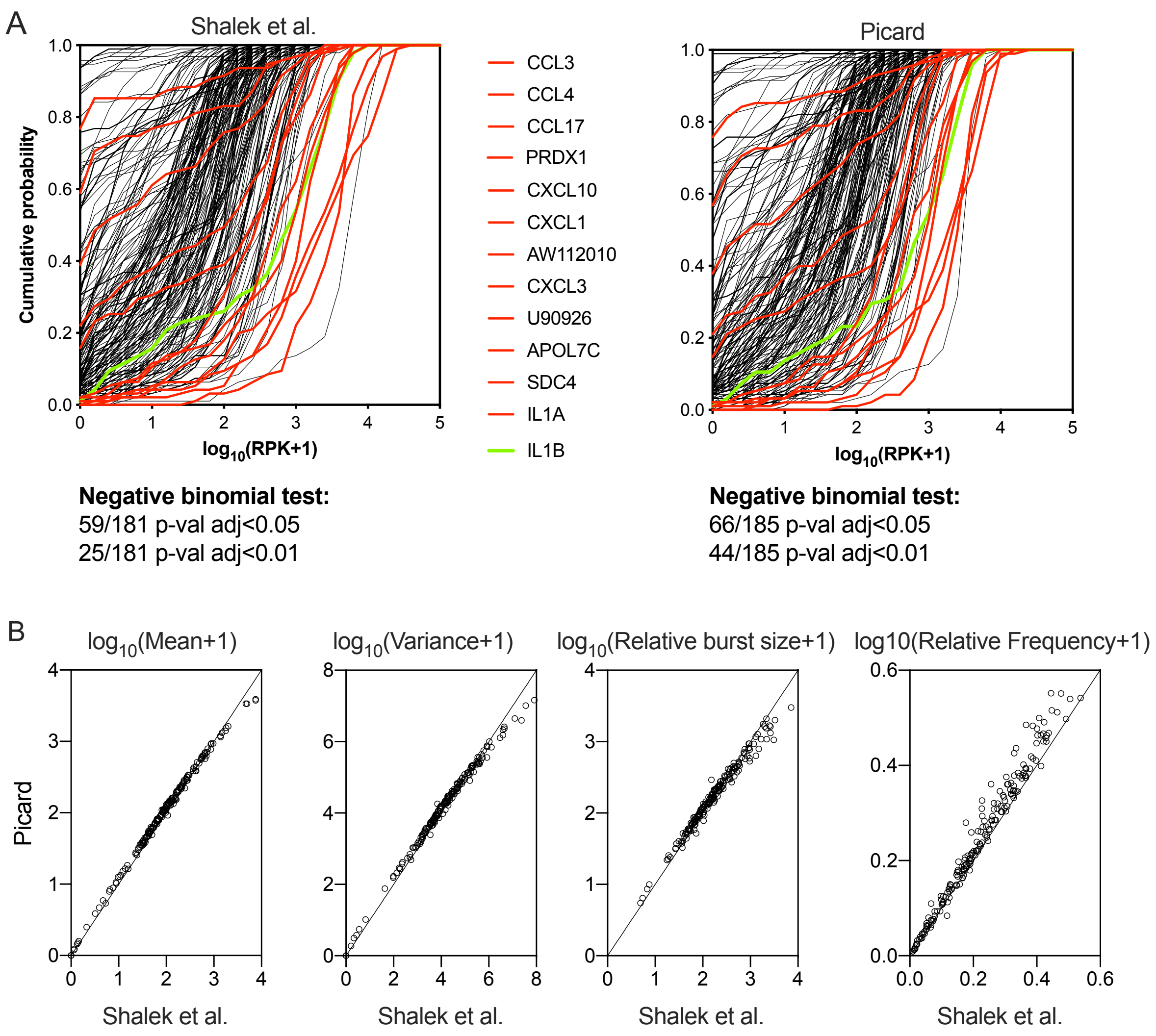

Figure S20. Validation of scRNA-seq measurements, related to Figure 4. A. Comparison between single cell distributions from (Shalek et al., 2014). LPS stimulation at 4h dataset and dataset obtained after re-mapping with Picard Tools (http://broadinstitute.github.io/picard/). Shown are cumulative distributions for 204 LPS-regulated genes from Fig. 3D, highlighted in red are high variability genes, in green *IL1β*. Shown are also fractions of genes (after removing low abundant genes), distribution of which does not fit negative binomial (as highlighted with chi-squared goodness-of-fit test p-values adjusted with Benjamini-Hochberg procedure for false discovery rate). **B.** Comparison between bursting characteristics for 204 LPS-regulated genes from A, in single cell distributions from (Shalek et al., 2014) and dataset obtained after re-mapping with Picard Tools. Characteristics presented in log_{10} scale, in black identity line.

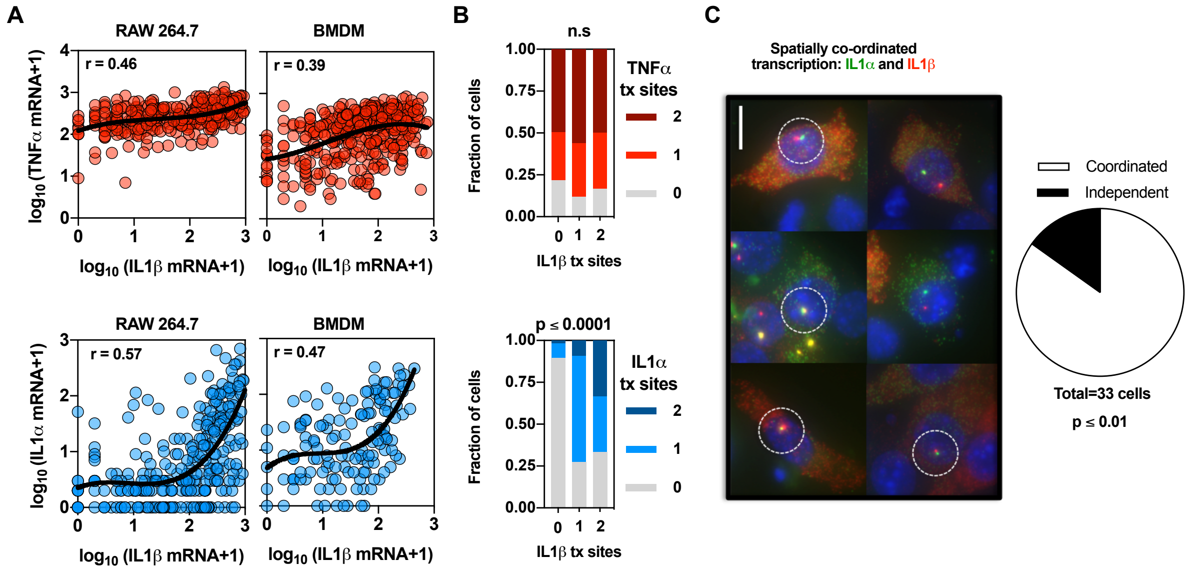

Figure S21. ***IL1α*** **and** ***IL1β*** **mRNA expression is coordinated in single cells at common transcription sites, related to Figure 5. A**. Correlation for *IL1β* vs. *TNFα* (top, in red) and *IL1β* vs. *IL1α* (bottom, in blue) mRNA counts. Shown are scatter plots of individual cell data (together with a cubic spline fit and a Spearman rank correlation *r*). RAW 264.7 and BMDM cells stimulated with 500 ng/ml of lipid A for 3 h. **B**. Correlation between transcription sites. Shown are the proportions of the co-activated transcription start sites for *IL1β* vs. *TNFα* (top) and *IL1β* vs. *IL1α* (bottom) for data in B. Shown also is a p-value for a chi-square test for the independence of Tx site occurrences between genes. **C**. Spatial analysis of *IL1α* and *IL1β* transcription. Deconvolved wide-field microscopy images of single cell smFISH mRNA counts for *IL1β* vs. *IL1α*. Circles indicate spatial coordination of transcription for data from A. Pie chart shows the proportion of spatially coordinated transcriptional sites for all cells in A exhibiting a common *IL1β* and *IL1α* Tx site (with a p-value for a chi-square test for independence).

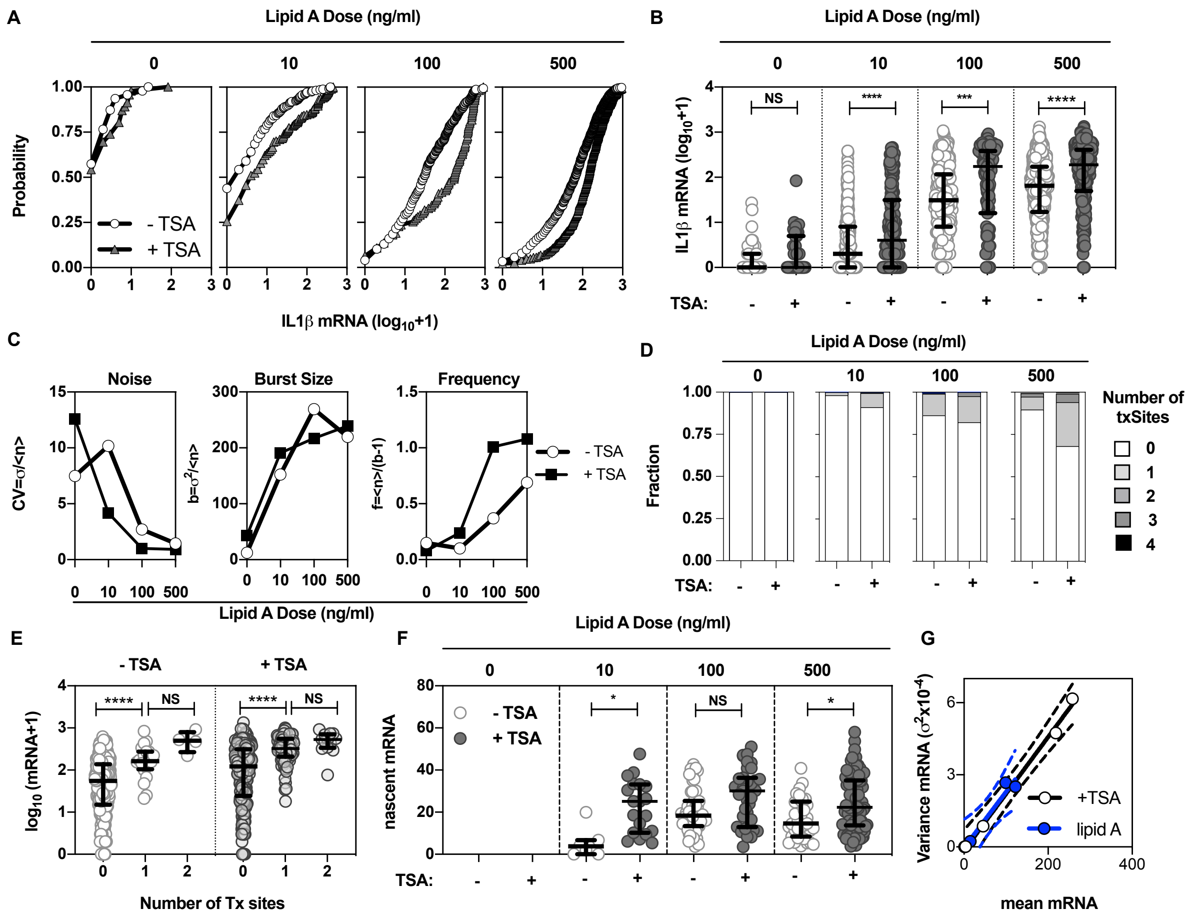

Figure S22. TSA modulation of *IL1β* mRNA distribution across lipid A dose-response, related to Figure 5. A. Shown are cumulative distribution plots of *IL1β* mRNA in BMDM cells pre-treated with TSA for 1 h (+TSA) or control group (-TSA) before stimulation with different lipid A doses for an additional 3 h. **B**. Individual cell mRNA counts data from A. Shown are data for 47, 276, 324 and 732 control (-TSA) as well as 46, 204, 110 and 305 (+TSA) for 0, 10, 100 and 500 ng/ml of lipid A, respectively. Results of the pairwise Mann-Whiney U tests summarised with ****- p-value <0.0001, ***- p-value <0.001, NS- not significant. **C**. Noise, burst size and frequency characteristics derived from data in B. **D**. Distribution of transcription sites observed in smFISH images from A. **E**. *IL1β* mRNA counts as a function of Tx site number for cells for 500 ng/ml lipid A dose. '*' denotes a statistical test (p-val<0.05) for one-way ANOVA with Tukey's correction for multiple comparisons. **F**. Quantification of nascent mRNA across lipid A dose-response from data in D. Shown is comparison across 0, 7, 52 and 35 (-TSA) as well as 0, 19, 35 and 114 (+TSA) transcription sites for 0, 10, 100 and 500 ng/ml of lipid A, respectively. Results of the pairwise nonparametric Mann-Whiney U tests summarised with ****- p-value <0.0001 and ***- p-value <0.001. **G.** Mean-variance relationship obtained for smFISH data for *IL1β* from A. Shown are the fitted regression lines

(with 95% confidence intervals in broken lines) together with individual data points. In blue cells pre-treated with TSA, in black lipid A treatment alone.

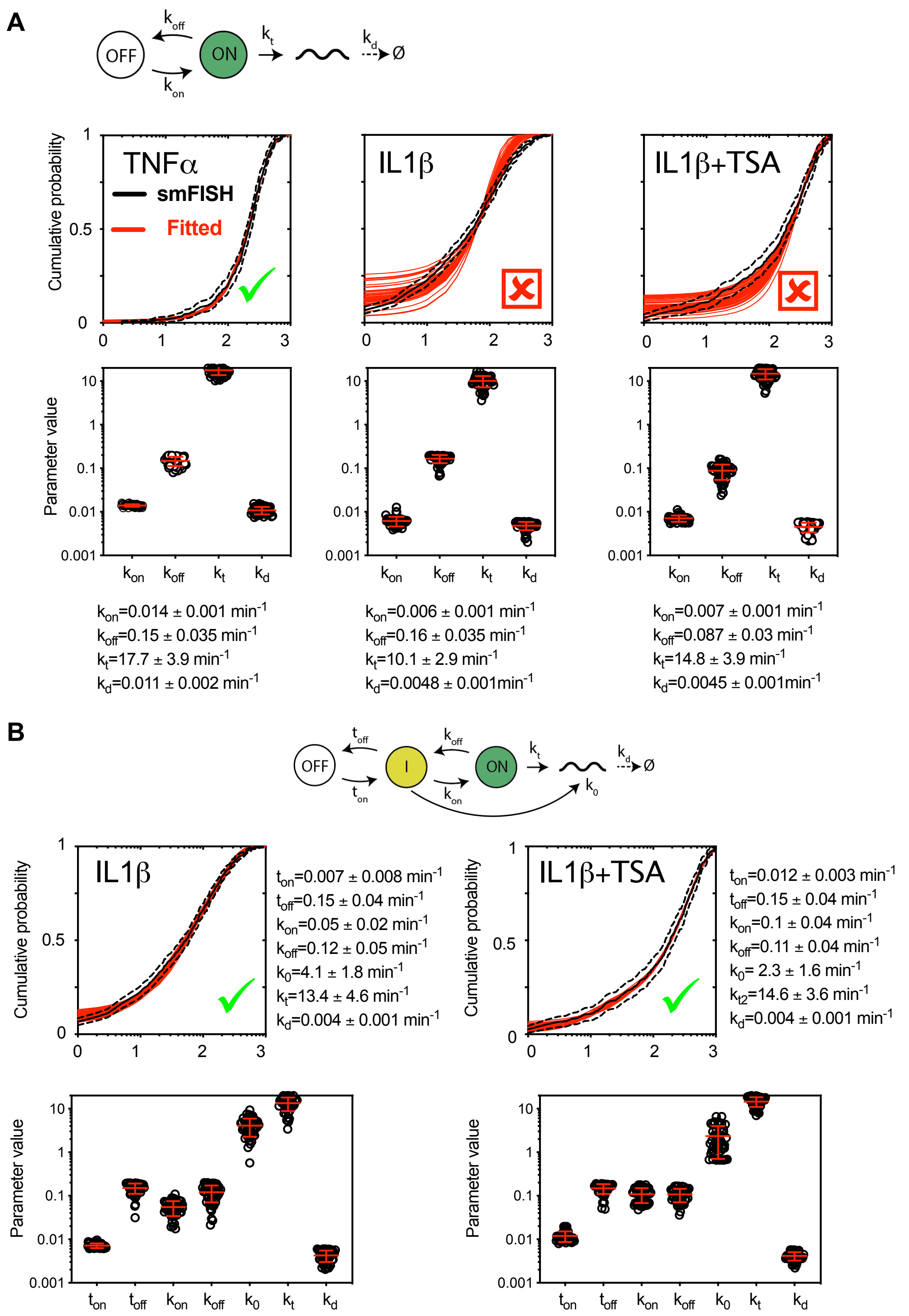

Figure S23. Characteristics of the fitted family of models for BMDM data, related to Figure 5. Distribution of the model parameters fitted to smFISH mRNA data at 3 h after 500 ng/ml lipid A stimulation in BMDM cells (in combination with TSA treatment, as in Fig. 5). Shown are parameters of 50 individual fits for one-step (**A**) and two-step (**B**) models summarised using a box plot (with mean ± SD in red). Fit quality summarised with green ticks and red crosses.
